# Efficacy and Safety of Natural Apigenin Treatment for Alzheimer's Disease: Focus on *In vivo* Research Advancements

**DOI:** 10.2174/1570159X23666241211095018

**Published:** 2024-12-11

**Authors:** Nan Zhang, Jianfei Nao, Xiaoyu Dong

**Affiliations:** 1 Department of Neurology, China Medical University Seventh Clinical College, Fushun, Liaoning P.R. China;; 2 Department of Neurology, Shengjing Hospital of China Medical University, Shenyang, Liaoning, P.R. China

**Keywords:** Alzheimer's disease, apigenin, meta-analysis, pharmacological mechanism, systematic review, apigenin treatment

## Abstract

**Background:**

Alzheimer's Disease (AD) is the most common dementia in clinics. Despite decades of progress in the study of the pathogenesis of AD, clinical treatment strategies for AD remain lacking. Apigenin, a natural flavonoid compound, is present in a variety of food and Chinese herbs and has been proposed to have a wide range of therapeutic effects on dementia.

**Objective:**

To clarify the relevant pharmacological mechanism and therapeutic effect of apigenin on animal models of AD.

**Methods:**

Computer-based searches of the PubMed, Cochrane Library, Embase, and Web of Science databases were used to identify preclinical literature on the use of apigenin for treating AD. All databases were searched from their respective inception dates until June 2023. The meta-analysis was performed with Review manager 5.4.1 and STATA 17.0.

**Results:**

Thirteen studies were eventually enrolled, which included 736 animals in total. Meta-analysis showed that apigenin had a positive effect on AD. Compared to controls, apigenin treatment reduced escape latency, increased the percentage of time spent in the target quadrant and the number of plateaus traversed; apigenin was effective in reducing nuclear factor kappa-B (NF-κB) p65 levels; apigenin effectively increased antioxidant molecules SOD and GSH-px and decreased oxidative index MDA; for ERK/CREB/BDNF pathway, apigenin effectively increased BDNF and pCREB molecules; additionally, apigenin effectively decreased caspase3 levels and the number of apoptotic cells in the hippocampus.

**Conclusion:**

The results show some efficacy of apigenin in the treatment of AD models. However, further clinical studies are needed to confirm the clinical efficacy of apigenin.

## 1 INTRODUCTION

Dementia is an acquired impairment of intellectual function and other cognitive skills that leads to a reduced ability to perform daily activities [1]. Alzheimer's disease (AD) is the most common dementia occurring in old age. It involves neurodegeneration of the central nervous system (CNS) and is characterized by progressive cognitive dysfunction and behavioral disorders [2]. Clinical manifestations of AD include memory impairment, agnosia, aphasia, impairment of visuospatial ability, and personality and behavior changes [3]. AD is the fourth leading cause of death in older people, after cancer, heart disease, and cerebrovascular disease, and it is the third most costly of all diseases. Consequently, it presents significant challenges to millions of patients, their families, and society as a whole [4]. It is estimated that more than 1.315 billion people worldwide will have AD by 2050 [5].

The pathogenesis of AD is complex and has not been fully deciphered [6]. Deposition of amyloid beta (Aβ) in the brain parenchyma and cerebral vessels, as well as the presence of neurofibrillary tangles (NFTs) and progressive loss of synapses, are central neuropathologic features of AD [7]. However, to date, it is not clear what primarily triggers and drives disease progression [8, 9]. In the last decade, despite the growing focus on AD research, the complex molecular mechanism underlying the pathophysiology of AD remains incompletely understood. Among the different multifactorial pathways affected by the disease, oxidative stress, harmful changes in mitochondria, reduced utilization of brain glucose, and neuroinflammation are currently considered to be important factors in disease initiation and/or progression [10].

Aducanumab, the first antibody approved for the treatment of AD by the Food and Drug Administration in nearly 20 years, sparked a marked controversy soon after its launch [11]. Clinical trials showed that the monoclonal drug cleared Aβ from the brain, but there was insufficient evidence that it slowed or stopped the progression of AD [12]. Additionally, approximately 40% of patients treated with this monoclonal antibody experienced severe side effects, including cerebral edema (ARIA-E) and hemorrhage (ARIA-H) [13]. Lecanemab, another Aβ antibody drug that was released after aducanumab, shares the same mechanism (both target Aβ), but the drugs have completely different epitopes on which they act [14]. The effect of lecanemab as compared to aducanumab is clear and is well-documented by clinical data [15]. In a phase 3 clinical trial, lecanemab reduced dementia-like amyloid and delayed early AD disease progression by 27% over 18 months of treatment, meeting the primary endpoint and all key secondary endpoints, with highly statistically significant results [16]. However, the potential side effects of drugs targeting Aβ, such as cerebral edema and hemorrhage, limit their clinical use. Currently, the central cholinesterase inhibitors donepezil and cabalatine and the glutamate receptor antagonist memantine are widely used in the treatment of AD [17]. Nevertheless, these existing therapeutic strategies can only relieve symptoms but cannot cure AD [18]. Therefore, the prognosis of the majority of dementia patients remains poor, and new therapeutic methods to cure this disease must be developed.

Natural compounds have become a hot topic in research because of their advantages, in particular, their convenience, high safety, low toxicity, and lack of side effects, which are unmatched by conventional Western drugs [19, 20]. A variety of natural compounds have been shown to play crucial roles in the treatment of chronic diseases [21]. Polyphenols are the most abundant and widely distributed substances in plants [22]. They share the common feature of an aromatic ring containing at least one hydroxyl substituent [23], and they are often linked to sugars (glycosides) and, therefore, tend to be water-soluble [24]. The largest group of naturally occurring polyphenols are flavonoids, such as apigenin [25].

**Fig. (1):**
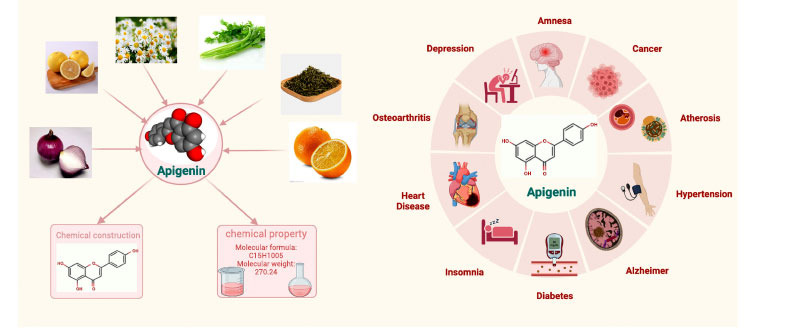
Main sources and map indicating the functions of apigenin.

Apigenin (4’,5,7-trihydroxyflavone) (AP) is a yellow crystalline substance with the molecular formula C_15_H_10_O_5_, a molecular weight of 270.24 Da, and a melting point of 347-348°C [26]. It is a flavonoid derivative with three hydroxyl substituents, which is insoluble in water but is soluble in organic solvents. It is a ligand of naturally occurring glycosides, and it is found mainly in its glycosylated forms in vegetables (parsley, celery, onion), fruits (oranges, lemons, grapefruit), herbs (chamomile, thyme, oregano, Rorer) and plant-based beverages (tea, beer, wine) [27]. Particularly high levels are found in parsley and celery: celery contains 108 mg/kg AP [28] (Fig. **1**).

It's delayed plasma clearance and slow decomposition in the liver increase its systemic bioavailability and make AP a potent therapeutic agent in drug research and development [29]. The solubility of AP in water is very low (1.35 μg/mL), although it has high permeability [30]; thus, the use of AP in *in vivo* studies may be limited. It has been shown that providing AP in liposomes, polymer micelles, and nanosuspension could improve its solubility and stability, as well as the dosage of AP, and can prolong the pharmacological activity of the compound [31-33].

Emerging studies have shown that AP has therapeutic effects on diabetes, depression, amnesia, osteoarthritis, insomnia, cancer, arteriosclerosis, hypertension, and heart disease [34-36]. Several studies have reported that AP improves memory and reduces amyloid deposition in animal models [37, 38]. Moreover, clinical trials have found that AP improves cognitive function and provides neurovascular protection [39]. The neuroprotective mechanisms of AP in AD involve reduction of Aβ protein deposition, inhibition of aggregation of hyperphosphorylated tau proteins into NFTs, reduction of neuroinflammation, anti-oxidative stress and anti-apoptotic effects, inhibition of nitrosylation, inhibition of acetylcholinesterase (AChE), restoration of histone acetylation, promotion of synapse formation, and regulation of the extracellular signal-regulated kinase (ERK)/cAMP-response element binding protein (CREB)/brain-derived neurotrophic factor (BDNF) pathway. The multi-mechanism and multi-target pharmacological characteristics of apigenin make it possible for apigenin to become a new therapeutic drug for dementia [40].

Thus, while AP has been shown to be effective against AD, the mechanism remains uncertain. Hence, to clarify the relevant pharmacological mechanism and therapeutic effects of AP, we conducted a systematic review and meta-analysis of data obtained from studies of animal models of AD.

## 2 MATERIALS AND METHODS

### 2.1 Search Strategy

This systematic evaluation and meta-analysis were performed according to the Preferred Reporting Items for Systematic Evaluations and Meta-Analyses (PRISMA) statement [41]. We performed electronic searches for relevant publications on the treatment of AD with AP in the PubMed, Cochrane Library, Embase, and Web of Science databases from their respective inception dates up to June 2023.

Literature was retrieved by combining free words with subject terms. The search terms included the following: apigenin, AD, Alzheimer's Disease, Alzheimer's, and Alzheimer’s disease. The full PubMed search is: “Alzheimer's Disease” [Title] OR “AD” [Title] OR “Alzheimer Disease” [Title] OR “Alzheimer Disease” [Title] OR “Alzheimer Disease” [MeSH Terms]) AND (“Apigenin” [MeSH Terms] OR “Apigenin” [Title/Abstract]. We considered all studies published that might be eligible for review. Moreover, the references included in the identified publications were further screened for additional reports.

We included *in vivo* studies, in which a control group was available and for which descriptive statistical results of clinical measurements were reported (*i.e*., mean, standard deviation, sample size of participating animals). We excluded publications that were reviewed, editorials, *in vitro* studies, conference papers, unrelated topics, investigated AP compatibility with other drugs, missing control groups, or that presented incomplete data.

The databases were screened, and information was extracted independently by two authors according to the above inclusion and exclusion criteria. Disagreements were resolved by discussion to consensus, or a third author assisted in the adjudication.

### 2.2 Data Extraction

The research data were extracted using a self-made data extraction table. The main content extracted was the following: (1) the basic information of the research, including the first author and the year of publication; (2) basic information of experimental animals, including breed, sex, sample size, and weight; (3) method for inducing an AD animal model; (4) detailed information on the intervention population, including dose administered, mode of administration and time of administration; (5) the outcome indicators; and (6) the main conclusions of the study. When multiple independent groups were included in a study (*e.g*., different drug doses), these data were extracted and treated as separate experiments, with the corresponding control groups also treated separately. Graph-based data were calculated using WebPlot Digitizer version 4.5 software (https://automeris.io/WebPlotDigitizer/).

### 2.3 Risk-of-bias Assessment of Included Studies

The risk-of-bias of the included studies was assessed by two authors using the modified SYRCLE risk-of-bias tool based on Cochrane tools [42]. The SYRCLE animal experiment risk-of-bias assessment tool included 10 items: (1) random sequence generation; (2) baseline characteristics; (3) allocation concealment; (4) random housing; (5) blinding of participants and personnel; (6) random outcome assessment; (7) blinding of outcome assessment; (8) incomplete outcome data; (9) selective outcome reporting; (10) other sources of bias. These were scored as “Low risk,” “Unclear,” and “High risk” for each indicator [43].

Two authors independently performed the risk-of-bias assessments and cross-checked the results. Cases of disagreement were resolved by discussion to consensus.

### 2.4 Statistical Analysis

The mean and standard deviation (SD) values of the recorded data were collected using WebPlot Digitizer 4.5 software. To improve the comparability of results of the same indicator tested by different methods, we evaluated the standardized mean differences (SMD) to eliminate the effect of magnitude between the experimental data. The meta-analysis was performed using a Review Manager 5.4.1. (https://training.cochrane.org/online-learning/core-software/revman) and STATA17.0 (https://www.stata.com/products/windows/). The chi-square test was used to analyze the heterogeneity of the results. If there were no statistical heterogeneity (*p* ≥ 0.1, I2 ≤ 50%), a fixed-effects model was used for meta-analysis. If there were statistical heterogeneity among the results (*p* < 0.1, I2 > 50%), a random-effects model was performed for meta-analysis. The summary statistics of outcomes were quantitatively determined using the SMD with corresponding 95% confidence intervals (95% CIs), with *p* < 0.05 indicating a statistically significant difference. When significant heterogeneity existed, sensitivity analyses were conducted by excluding the included data on a case-by-case basis to assess the stability of the findings or subgroup analyses were performed to explore potential sources of heterogeneity in the main validated outcome indicators further. To determine whether publication bias existed among the included studies, we constructed funnel plots of the main outcome indicators. We assessed funnel plot asymmetry using the Egger test and defined significant publication bias as *p* < 0.05. For the primary outcome indicators, sensitivity analyses were performed using Stata 15.0 software when there was significant heterogeneity, and sensitivity analyses were performed to determine whether these changes would affect the combined outcome estimates by excluding the included data one by one. A meta-regression analysis was then performed to identify confounding factors that might contribute to high heterogeneity. Finally, subgroup analyses were performed on the primary outcome indicators of escape latency and target quadrant dwell time to further explore potential sources of heterogeneity in the primary validated outcome indicators and to explore the impact of different experimental elements on the results to facilitate translation to clinical trials. Subgroup analysis was performed according to the following characteristics: AP dose (≥ 50 mg/kg or < 50 mg/kg) and intervention mode (intraperitoneal [*i.p*] or transoral administration [*oral*]). Sources of publication bias were analyzed using funnel plots and Egger's test, and *p* < 0.05 was considered statistically significant. When the presence of publication bias was indicated, trim and fill analysis was used to evaluate the effect of publication bias on the results, and if the combined effect size estimates did not change significantly, this indicated that the effect of publication bias was not significant, and the results were more robust.

## 3 RESULTS

A total of 336 articles were identified using our retrieval strategy with the stated inclusion and exclusion criteria. After screening the titles, abstracts, and full texts of the articles, 13 eligible studies, including 736 animals, were finally included. These studies evaluated the neuroprotective effects of AP in animal models of AD and explored possible therapeutic mechanisms. Duplicates were first removed using Endnote software, and then the remaining duplicates were manually removed, resulting in an initial screening of 291 articles. By reading the titles, abstracts and full text, screening was performed according to inclusion and exclusion criteria. One hundred fifty-four were excluded because they were review studies, 44 studies were excluded because they were *in vitro* studies, 5 studies were excluded because they were editorials, 16 studies were excluded because they were conference papers, 25 studies were excluded because they were unrelated topics, 16 studies were excluded because they lacked controls, 11 studies were excluded because data were incomplete and 7 studies were excluded because AP was paired with other drugs, resulting in the inclusion of 13 studies, all of which were published after 2010 and 6 of which were published in the last 3 years, suggesting that the protective effect of AP on AD has recently received increasing attention. The literature screening process and results are shown in Fig. (**2**).

### 3.1 Characteristics of the Included Studies

Of the 13 included studies, 3 studies [44-46] used a lateral ventricular injection of Aβ25-35 for induction, 1 study [47] used a lateral ventricular injection of Aβ1-42 induction model, 1 [37] used an amyloid-beta precursor protein (APP)/ presenilin-1 (PS1) transgenic model, 1 [48] used bilateral oophorectomy, and the remaining 7 studies were chemically induced using lipopolysaccharide (LPS) [49], isoacteoside (ISO) [50], scopolamine (SCOP) [51], streptozocin (STZ) [52], D-galactose [53], methotrexate (MTX) [54], and acetonitrile (ACN) [55]. The following species were used in the studies: Swiss albino mouse, Wistar rat, SD rat, Institute of Cancer Research (ICR) mice, Kunming mouse, B6C3-Tg mice and wild-type mice. The number of animals used in the included literature ranged from 3 to 20 animals per group. Most of the studies were based on body weight, with AP doses of 10, 20, 25, 40, 50, 100, 117, 234, and 351 mg/kg, and one study with 2 mg and dosing durations ranging from 7 days to 12 weeks. The most used animal model was the Aβ25-35 induction model, and the most involved routes of administration in the studies were transoral administration and intraperitoneal injection. The outcome assays involved in the study included behavioral assays (Morris Water Maze (MWM) escape latency, the percentage of residence time in target quadrant, the number of crossings in the right quadrant), inflammatory indicators nuclear factor kappa-β (NF-κB) p65 protein, ERK/CREB/BDNF pathway-related molecules (BDNF and pCREB), oxidative stress indicators, including antioxidant enzymes superoxide dismutase (SOD), glutathione peroxidase (GSH-Px) and oxidative damage-related product malondialdehyde (MDA), apoptosis indicators, cysteinyl aspartate specific proteinase-3 (Caspase-3) and apoptotic neurons in the hippocampus. Given that most studies performed MWM escape latency and the percentage of residence time in target quadrant, and that the MWM is a very versatile behavioral experiment for animal models of AD, using it as a primary outcome indicator also reduces the heterogeneity between studies caused by different experimental protocols (Tables **1** and **2**).

### 3.2 Risk of Bias Assessment Results

**Fig. (3):**
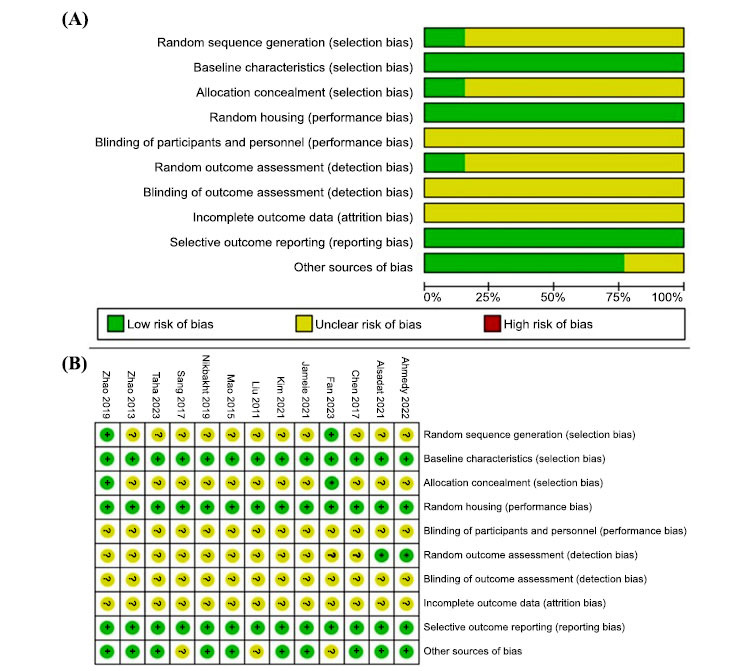
(**A**) Table based on SYRCLE document quality assessment. (**B**) Graph-based on SYRCLE document quality assessment.

The 13 included studies had a methodological quality score of 3-6 based on the SYRCLE risk of bias tool (Fig. **3**). There were 2 studies [47, 55] that described in detail the generation of random sequences (both random number table methods) so that random sequences and allocation of hidden risks were classified as low risk. All studies described the animal characteristics in detail and ensured similarity between animals. Therefore, the risk of baseline characteristics was all low. Thirteen studies stated that animals were housed in a standardized laboratory environment controlling for the same temperature and humidity; therefore, the risk of animal placement randomization was low. All studies did not mention blinding of participants and study personnel or outcome evaluators; therefore, the risk of bias was assessed as unclear; 2 studies [44, 49] stated that animals were randomly selected for outcome assessment, and we considered the likelihood of bias to be low and the remaining 11 studies to be unclear; none of the studies mentioned whether data were missing, so the risk of completeness of outcome data was rated as unclear; all studies adequately reported the prior stated variables, and there were no variables that were not stated, so the risk of selective reporting was categorized as low; 10 studies stated no potential conflict of interest, and we classified the other risk entries as low risk.

### 3.3 Intervention Effects

Cognitive dysfunction is an important clinical manifestation of AD, and the assessment of memory and executive function is an important part of cognitive function assessment in AD. The MWM is a spatial learning test for rodents that relies on distal cues to navigate from a starting location around the perimeter of an open swimming pool to determine the location of an underwater escape platform and is one of the most commonly used behavioral tests for spatial learning and long-term spatial memory in experimental animals [56]. Activation of the CREB-BDNF signaling pathway in the brain is a potential therapeutic target for the treatment of cognitive disorders such as AD [57, 58]. In recent years, several studies have reported that activation of the ERK/ CREB/BDNF pathway would help to improve AD [59]. Oxidative stress is an early event in the onset and development of AD [60]. The extent of oxidative damage can be determined by assessing the activity of antioxidant enzymes (*e.g*., SOD, GSH-Px) and the levels of oxidative damage-related products (*e.g*., MDA, a key indirect indicator of reactive oxygen species (ROS) production) [61]. Apoptosis is undoubtedly an important pathway for the loss of pyramidal neurons, leading to cognitive dysfunction [62, 63]. The inflammatory response of the brain is an important feature of AD and plays an important role in the pathogenesis of AD, and NF-κB has an important regulatory role in the inflammatory response [64]. The MWM has become one of the “gold standards” in behavioral neuroscience, and because most of the included studies measured MWM escape latency and the percentage of residence time in the target quadrant, we used these as primary outcome measures, with neurobiochemical changes as secondary outcome measures.

#### 3.3.1 Morris Water Maze

##### 3.3.1.1 Escape Latency

**Fig. (4):**
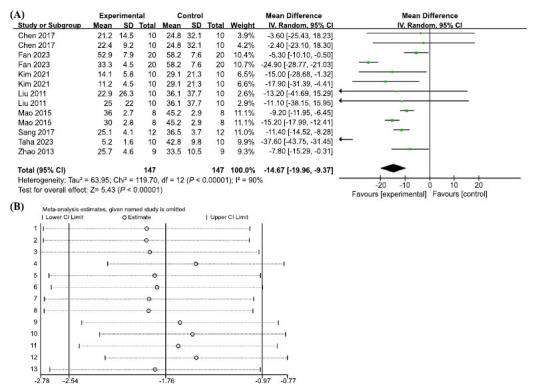
(**A**) Meta-analysis forest plot comparing apigenin *versus* vehicle treatment. Outcome: Morris water maze, escape latency (sec). (**B**) Sensitivity analysis demonstrated the reliability of AP to affect escape latency.

In terms of escape latency in the MWM test, 13 groups of trials from 8 included studies demonstrated high heterogeneity (I2 = 90%, *p <* 0.00001); therefore, a random-effects model was used. The results showed that AP treatment significantly reduced the time required to escape from the MWM platform and improved the learning and memory abilities of the animals, with statistically significant differences (MD = -14.67, 95% CI: (-19.96, -9.37), *p* < 0.00001) (Fig. **4A**). To explore the sources of heterogeneity, we performed sensitivity analyses, excluding one study at a time from the original analysis, with no significant between-study heterogeneity, demonstrating that our primary results were robust (Fig. **4B**). A meta-regression was performed using dose mode of administration as a categorical feature to detect sources of between-trial heterogeneity. The meta-regression analysis suggested that *p* > 0.05 (Table **3A**) and no source of heterogeneity was found, suggesting that neither dose nor mode of administration was responsible for the high heterogeneity.

##### 3.3.1.2 Percentage of Residence Time in the Target Quadrant

**Fig. (5):**
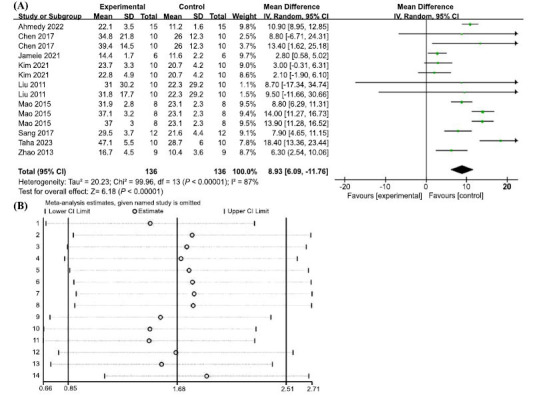
(**A**) Meta-analysis forest plot comparing apigenin *versus* vehicle treatment. Outcome: Morris water maze, the percentage of residence time in the target quadrant. (**B**) Sensitivity analysis demonstrated the reliability of AP in affecting the percentage of residence time in the target quadrant.

Fourteen sets of trials from the nine included studies showed high heterogeneity (I2 = 87%, *p* < 0.00001), therefore a random effects model was used. AP treatment significantly increased the percentage of residence time in the target quadrant, with a statistically significant difference (MD = 8.93, 95% CI: (6.09, 11.76, *p* < 0.00001) (Fig. **5A**). To explore the reasons for the heterogeneity, further sensitivity analysis was performed, and the heterogeneity did not change significantly, demonstrating robust results (Fig. **5B**). A further meta-regression analysis was performed to detect the source of heterogeneity between trials by using dose and mode of administration as categorical features, and the results suggested that *p* > 0.05 (Table **3B**), no source of heterogeneity was found, suggesting that neither dose nor mode of administration was responsible for the high heterogeneity.

##### 3.3.1.3 Number of Crossings in the Right Quadrant

**Fig. (6):**
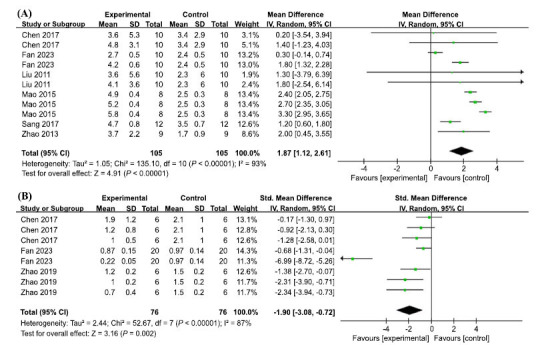
(**A**) Meta-analysis forest plot comparing apigenin *versus* vehicle treatment. Outcome: Morris water maze, the number of crossings in the right quadrant. (**B**) Meta-analysis forest plot comparing apigenin *versus* vehicle treatment. Outcome: NF-κB.

Eleven sets of trials from the six included studies showed high heterogeneity (I2 = 93%, *p* < 0.00001); therefore, a random effects model was used. AP showed strong efficacy in increasing the number of crossings in the right quadrant, with a statistically significant difference (MD = 1.87, 95% CI: (1.12, 2.61), *p* < 0.00001) (Fig. **6A**).

#### 3.3.2 NF-κB

The NF-κB protein is one of the key mediators of neuroinflammation, and cells secrete many typical inflammatory cytokines such as interleukin-1β (IL-1β), IL-6 and tumor necrosis factor-α (TNF-α) through the activation of NF-κB pathway, which can directly damage neurons in the brain [65]. A meta-analysis of eight sets of trials from the three included studies revealed a large heterogeneity (I2 = 87%, *p* < 0.00001) and therefore, a random effects model was used. AP treatment significantly reduced NF-κB p65 levels (SMD = -1.9, 95% CI: (-3.08, -0.72), *P* = 0.002) (Fig. **6B**).

#### 3.3.3 ERK/CREB/BDNF Pathway

##### 3.3.3.1 BDNF

**Fig. (7):**
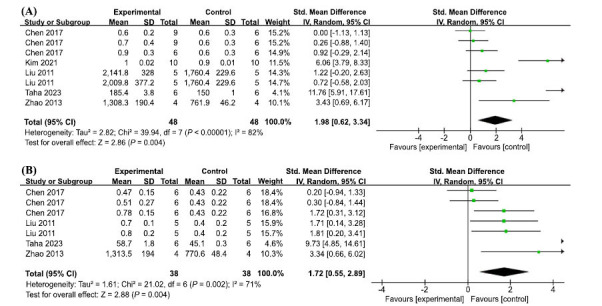
(**A**) Meta-analysis forest plot comparing apigenin *versus* vehicle treatment. Outcome: BDNF levels. (**B**) Meta-analysis forest plot comparing apigenin *versus* vehicle treatment. Outcome: pCREB levels.

BDNF, a member of a family of growth factors found in the brain and central nervous system, regulates processes such as neuronal growth differentiation and synapse formation [66]. A meta-analysis of eight sets of trials from five studies showed significant heterogeneity (I2 = 82%, *p* < 0.00001); therefore, we used a random effects model. The results showed that AP treatment significantly increased BDNF content (SMD = 1.98, 95% CI: (0.62, 3.34), *P* = 0.004) (Fig. **7A**).

##### 3.3.3.2 pCREB

CREB is a nuclear transcription factor that is essential for hippocampus-dependent memory, and CREB activation phosphorylation is required for its function [67]. A meta-analysis of 7 groups from 4 studies showed high heterogeneity (I2 = 71%, *P* = 0.002); therefore, a random effects model was used. We found that AP treatment significantly increased pCREB levels (SMD = 1.72, 95% CI: (0.55, 2.89), *P* = 0.004) (Fig. **7B**).

#### 3.3.4 Oxidative Stress Indicators

##### 3.3.4.1 SOD

**Fig. (8):**
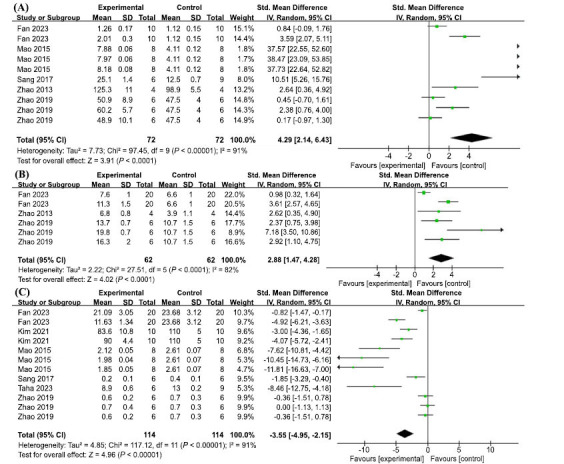
(**A**) Meta-analysis forest plot comparing apigenin *versus* vehicle treatment. Outcome: SOD levels. (**B**) Meta-analysis forest plot comparing apigenin *versus* vehicle treatment. Outcome: GSH-px levels. (**C**) Meta-analysis forest plot comparing apigenin *versus* vehicle treatment. Outcome: MDA levels.

Oxidative stress is an imbalance between the production and scavenging of oxygen free radicals in the body or cells, resulting in oxidative damage to the body or cells. SOD is an antioxidant enzyme that exists in the body itself, and as an important component of the enzymatic antioxidant system, it plays an irreplaceable role in balancing the oxidative stress response in the body [68]. The results of the five studies included in the study are as follows: Ten sets of trials from the five included studies showed high heterogeneity (I2 = 91%, *p* < 0.00001) and therefore, a random effects model was used. AP treatment significantly increased SOD levels (SMD = 4.29, 95% CI: (2.14, 6.43), *p* < 0.00001) (Fig. **8A**).

##### 3.3.4.2 GSH-Px

GSH-Px is an important peroxidolytic enzyme *in vivo* that reduces peroxides and protects cells from oxidative damage. Six sets of trials from the three included studies showed high heterogeneity (I2 = 82%, *p* < 0.0001); therefore, a random effects model was used. AP treatment significantly increased GSH-px levels (SMD = 2.88, 95% CI: (1.47, 4.28), *p* < 0.0001) (Fig. **8B**).

##### 3.3.4.3 MDA

MDA is one of the products formed by the reaction of lipids with oxygen free radicals, and its content represents the degree of lipid peroxidation. Twelve sets of trials from the six included studies showed high heterogeneity (I2 = 91%, *p* < 0.00001); therefore, a random effects model was used. AP treatment significantly reduced MDA (SMD =-3.55, 95% CI: (-4.95, -2.15), *p* < 0.00001) (Fig. **8C**).

#### 3.3.5 Apoptosis Indicators

##### 3.3.5.1 Caspase 3

**Fig. (9):**
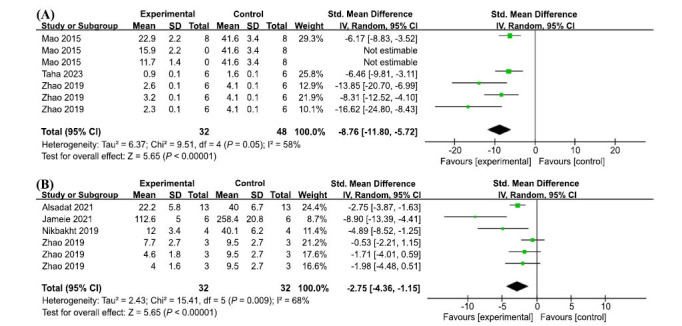
(**A**) Meta-analysis forest plot comparing apigenin *versus* vehicle treatment. Outcome: caspase 3 levels. (**B**) Meta-analysis forest plot comparing apigenin *versus* vehicle treatment. Outcome: Apoptotic neurons in the hippocampus.

The Caspase family plays a central role in the control of apoptosis, and Caspase 3 of the family is a key execution molecule in the apoptotic process [69]. A meta-analysis of six groups from three studies showed significant heterogeneity (I2 = 58%, *P* = 0.05), so a random effects model was used. The results showed that AP treatment significantly reduced caspase 3 levels (SMD = -8.76, 95% CI: (-11.80,-5.72), *p* < 0.00001) (Fig. **9A**).

##### 3.3.5.2 Apoptotic Neurons in the Hippocampus

Damage to hippocampal neurogenesis plays a role in memory deficits associated with AD by reducing the availability of immature neurons for memory formation [70]. A meta-analysis of six groups from the four included studies showed significant heterogeneity (I2 = 68%, *P* = 0.009), so a random effects model was used. AP treatment significantly reduced the number of apoptotic and necrotic cells (SMD =-2.75, 95% CI: (-4.36, -1.15), *P* = 0.0008) (Fig. **9B**).

### 3.4 Subgroup Analysis

To investigate the effect of different subgroup factors on outcome indicators, we performed subgroup analyses of the main outcome indicators using the dose administered and the mode of administration as categorical features:

#### 3.4.1 Escape Latency

##### 3.4.1.1 Intervention Dose

**Fig. (10):**
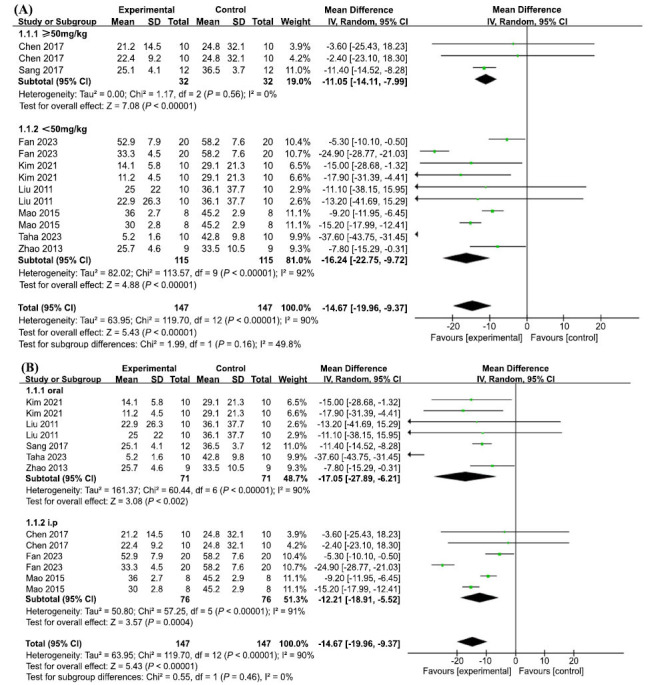
Subgroup analyses of the escape latency. (**A**) The dose of intervention on the effect size of the outcome measure; (**B**) The methods of intervention on the effect size of the outcome measure.

Intervention doses were divided into two groups of ≥ 50 mg/kg and < 50 mg/kg. The results suggested that the variability within subgroups was not significantly reduced, and the test for subgroup differences showed no statistically significant subgroup effect (*P* = 0.16), suggesting that the intervention dose did not affect the effect of escape latency (Fig. **10A**).

##### 3.4.1.2 Intervention Methods

The intervention methods were divided into two groups: *i.p* and *oral*. The results suggest that the variability within subgroups was not significantly lower, and tests for subgroup differences showed no statistically significant subgroup effects (*P* = 0.46), suggesting that the intervention modality was again not responsible for the high heterogeneity (Fig. **10B**).

#### 3.4.2 Percentage of Time in the Target Quadrant

##### 3.4.2.1 Intervention Dose

**Fig. (11):**
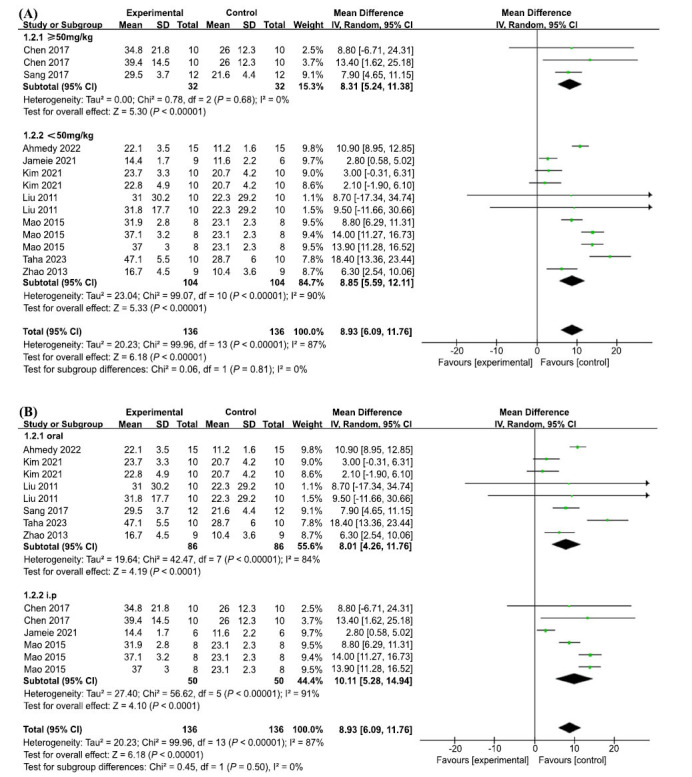
Subgroup analyses of the percentage of residence time in the target quadrant. (**A**) The dose of intervention on the effect size of the outcome measure; (**B**) The methods of intervention on the effect size of the outcome measure.

Intervention doses were divided into two groups: ≥ 80 mg/kg and < 80 mg/kg. The results suggested that the variability within subgroups was not significantly reduced, and the test for subgroup differences showed no statistically significant subgroup effect (*P* = 0.81), suggesting that the intervention dose did not affect the percentage of time in the target quadrant (Fig. **11A**).

##### 3.4.2.2 Intervention Methods

The intervention methods were divided into two groups: *i.p* and *oral*. The results suggest that the variability within subgroups was not significantly lower, and tests for subgroup differences showed no statistically significant subgroup effects (*P* = 0.5), suggesting that the intervention modality was again not responsible for the high heterogeneity (Fig. **11B**).

### 3.5 Publication Bias

Funnel plots and Egger plots were used to test for publication bias in the main outcome indicators of escape latency and target quadrant time.

#### 3.5.1 Escape Incubation Period

**Fig. (12):**
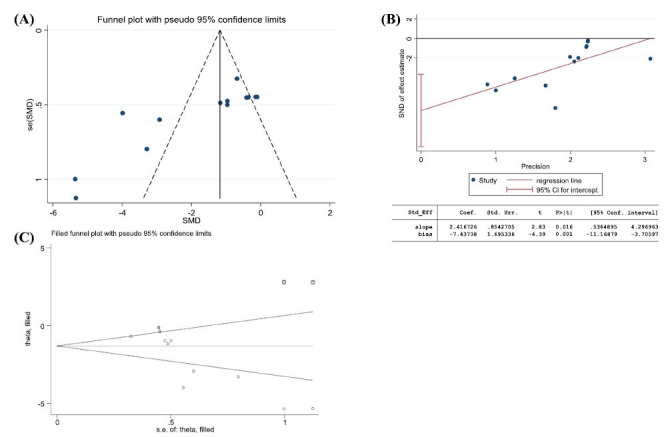
Escape latency. (**A**) Funnel plot for assessing potential publication bias; (**B**) Egger’s funnel plot analysis revealed potential publication bias; (**C**) The trim and fill analysis was used to evaluate the robustness of the results.

An asymmetric funnel plot was shown, suggesting a potential publication bias (Fig. **12A**). Further testing for publication bias using Egger, the outcome index *P* = 0.001, with an absolute value less than 0.05, indicates that publication bias does exist (Fig. **12B**). However, a further cut-and-complement test with insignificant changes in the combined effect size estimates indicated that the effect of publication bias was not significant, and the results were more robust (Fig. **12C**).

#### 3.5.2 Percentage of Time in the Target Quadrant

**Fig. (13):**
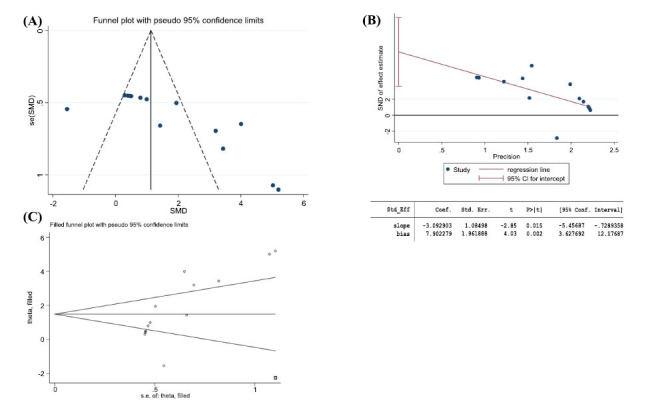
Percentage of residence time in target quadrant. (**A**) Funnel plot for assessing potential publication bias; (**B**) Egger’s funnel plot analysis revealed potential publication bias; (**C**) The trim and fill analysis was used to evaluate the robustness of the results.

An asymmetric funnel plot was shown, suggesting a potential publication bias (Fig. **13A**). Further testing for publication bias using Egger, the outcome index *P* = 0.002, with an absolute value less than 0.05, indicates that publication bias does exist (Fig. **13B**). However, a further cut-and-patch test with insignificant changes in the combined effect size estimates indicated that the effect of publication bias was not significant, and the results were more robust (Fig. **13C**).

## 4 DISCUSSION

**Fig. (14):**
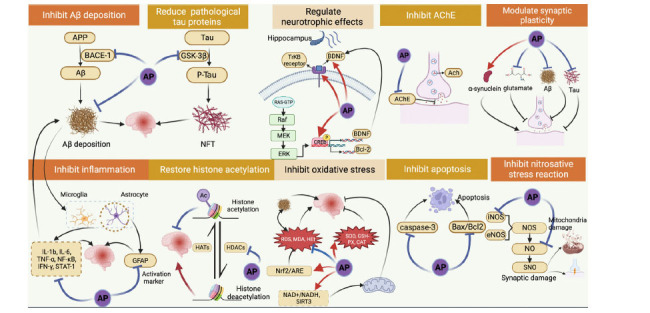
Possible mechanism of AP in the treatment of AD: inhibit BACE1 activity, reduce Aβ aggregation and deposition; inhibit GSK-3β to lower p-tau and reduce NFTs production; reduce glial cell activation, inhibit TLR4/NF-κB signaling pathway and reduce inflammatory factor release; improve mitochondrial dysfunction, activate NRF2 signaling pathway, increase antioxidant enzyme expression, reduce intracellular ROS levels and inhibit oxidative stress; increase Bcl2/Bax, downregulate apoptotic factors such as cytochrome C, Bax, caspase 9 and caspase 3 to exert anti-apoptotic effects; upregulate BDNF and its receptors TRKB and pCREB to restore the neurotrophic pathway ERK/CREB/BDNF; increase SYNI expression, inhibit glutamate release and promote synapse formation; inhibit HDAC activity and restores histone acetylation; inhibits AChE activity and increases cortical Ach levels; inhibits NO production and reduces nitrosylation.

AP has been found to be effective in the treatment of AD in previous studies, but the mechanism has not yet been fully elucidated (Fig. **14**). We conducted a meta-analysis of animal studies on this topic with a view to providing a pre-clinical evidence chain and rigorous, scientific, and systematic support for further clinical studies. To develop a more complete and rigorous chain of evidence, we reviewed the literature related to AP and AD extensively and summarized the possible mechanisms as follows.

### 4.1 Inhibition of Aβ Deposition

Aβ is one of the main constituents of senile plaques in the brain [71], which is the product of the cleavage of amyloid-beta precursor protein (APP) by a membrane protease [72]. Aβ40 and Aβ42 are two major forms of Aβ, and therapeutic strategies that reduce Aβ42 levels are associated with reduced AD risk, while Aβ40 is associated with enhanced neurotoxicity of Aβ42 [73]. Aβ impairs normal synaptic function and cognitive ability [74]; reduces levels of neurotrophic factors (NT), including nerve growth factor (NGF), BDNF, NT3, and NT4/5 [75]; and inhibits molecular transduction of learning and memory, including that which is related to mitogen-activated protein kinase (MAPK) and CREB activity [76].

APP cleaving enzyme 1 (BACE1) is an important aspartyl protease that is involved in the generation of myelin in peripheral nerve cells and contains two active-site transmembrane proteins that can form dimers outside of the cell [77]. Studies in transgenic mice have shown that a decrease in BACE1 prevents the production of Aβ [78]. In addition to its function as an Aβ-secretase, *i.e*., production of Aβ, BACE1 contributes to the typical memory and cognitive deficits of AD by regulating the cAMP/ Protein Kinase A (PKA)/CREB pathway, which is important in memory function [79]. Thus, it appears that BACE1 is an important factor in the treatment of AD and is the rate-limiting step in the amyloidogenic pathway [80].

Multiple studies have shown that AP improves memory and learning deficits by inhibiting Aβ aggregation and reducing Aβ amyloid deposition in AP-treated AD model mice. Jameie *et al.* suggested that AP significantly reduced the number of Aβ plaques in the hippocampus, as well as the symptoms of memory impairment, and decreased caspase 3 expression in ovariectomized animals [48]. AP inhibited the formation of Aβ42 aggregates and effectively alleviated the AD-like symptoms in transgenic *Drosophila* models of AD [81]. Furthermore, Zhao *et al.* found that AP played a crucial role in reducing primary Aβ levels and preventing Aβ accumulation in different regions of the brain [82]. Kim *et al.* indicated that AP down-regulated BACE1, along with PS1 and PS2 protein levels, improving cognitive and memory functions by regulating amyloidogenesis [51]. It has also been shown to affect APP processing and to modulate the amyloid production pathway, thereby attenuating Aβ deposition, reducing insoluble Aβ levels, and attenuating amyloid neurotoxicity [37, 38].

### 4.2 Inhibition of the Aggregation of Hyperphosphorylated Tau

NFTs consist of insoluble hyperphosphorylated tau protein and are regarded as another pathological hallmark of AD [83]. Tau protein is a microtubule-associated protein that is essential for the physiological function of axons and plays a key role in intracellular transport [84]. In AD, due to abnormal hyperphosphorylation, the tau protein loses its ability to bind to microtubules, resulting in abnormal aggregation into fibrous structures within neurons [85, 86]. Several recent observational studies have shown that p-tau concentrations are closely associated with Aβ deposition [87]. Aβ toxicity can be activated by tau, and hyperphosphorylation of tau produces NFTs [88].

Increased glycogen synthase kinase 3 (GSK3) activity was found in the brains of AD patients [89]. GSK3β is a key tau kinase in the brain that promotes the formation of NFTs in the helix [90]. Alsadat *et al.* found that AP (50 mg/kg) specifically inhibited GSK-3β and significantly reduced the level of hyperphosphorylated tau protein in the hippocampus of AD rat models by decreasing the expression of GSK3β, suggesting that AP is an ideal GSK-3β inhibitor for the treatment of AD [44].

### 4.3 Anti-inflammatory Effects

Inflammation clearly occurs in pathologically vulnerable areas of the AD brain, and it is associated with the complexity of local peri-inflammatory responses [91]. In AD, damaged neurons and neurites in the brain, as well as highly insoluble Aβ deposits and NTFs, elicit inflammation [92]. Microglia, astrocytes, the complement system, and inflammatory cytokines play important roles in the AD inflammatory response [93]. Aβ activates astrocytes and microglia, which subsequently activate a variety of inflammatory mediators, such as inflammatory cytokines (typically IL-1β, IL-6 and TNF-α), NF-κB, chemokines, interferon (IFN), and transforming growth factor β(TGF-β) [94]. These inflammatory mediators directly/indirectly damage neurons in the brain and induce excessive aggregation of Aβ, creating a vicious cycle of escalating impairment [95]. Several studies have reported the anti-inflammatory effects of AP. AP can significantly attenuate the activation of toll-like receptor 4 (TLR4)/NF-κB signaling pathway by decreasing nuclear translocation of activated NF-κB p65, thereby reducing the levels of IL-6 and TNF-α, inhibiting mitochondria-mediated neuronal apoptosis, and suppressing the inflammatory response [40, 55]. AP exhibited anti-inflammatory activity in rat models of subarachnoid hemorrhage and spinal cord injury, demonstrating neuroprotective effects by reducing the levels of inflammatory factors in neurological disorders [96, 97]. Mice fed a celery-based diet rich in AP (aglycone form) demonstrate improved absorption of AP and anti-inflammatory activity [98]. Rezai-Zadeh *et al.* demonstrated that AP could inhibit IFN-γ-induced signal transduction and transcriptional activator 1 (STAT1) phosphorylation in mouse microglia, which in turn inhibits the production of CD40, TNF-α, and IL-6 [99].

Astrocytes are the most widely distributed type of glial cells in the brain [100]. They support and protect neurons, promote neuronal repair, and improve the regulation of neurotransmitters [101]. Glial fibrillary acidic protein (GFAP) is a marker of astrocyte activation. When neurons are damaged, astrocytes are activated, producing large amounts of GFAP [102]. Liang *et al.* investigated the therapeutic effects of AP on neuroinflammation in mice with GFAP-IL6 expression by immunohistochemistry and behavioral experiments. Histological staining showed that AP reduced the number of activated glia in the cerebellum and hippocampus of GFAP-IL6 mice by about 30% and 25%, respectively [103]. Che *et al.* suggested that AP inhibits IL-31 and IL-33 production in microglial cells at the gene expression and secretion levels. Further analysis showed that AP can block nuclear translocation of activated NF-κB and STAT3, thereby blocking their DNA-binding activity, indicating the effects of AP on these two important inflammatory cytokines [104].

### 4.4 Anti-oxidative Stress

Emerging evidence suggests that accumulated oxidative stress may be one of the key mechanisms causing cognitive aging and neurodegenerative diseases, such as AD [105, 106]. Developing superoxide can clearly be seen in the hippocampus of rats treated with Aβ25-35, suggesting that oxidative stress is involved in Aβ25-35-induced neurodegeneration [107]. Mitochondrial fusion/fission has been found to be associated with defects in neuronal development, plasticity, and function, both *in vitro* and *in vivo* [108]. Mitochondria act as important regulators of cellular Ca^2+^ and play a crucial role in ensuring an adequate energy supply to neurons, as well as in functional axonal transport, by sequestering and releasing Ca^2+^ [109]. The accumulation of Aβ in mitochondria is one of the earliest pathological conditions in AD. AP ameliorates AD symptoms by improving mitochondrial dysfunction [46]. ROS are common by-products of inner mitochondrial membrane electron leakage during mitochondrial oxidative phosphorylation [110]. Under normal conditions, ROS are rapidly eliminated by enzymes, but when the mitochondria are perturbed, ROS production may exceed the cell's ability to neutralize these compounds, leading to oxidative damage [111]. Excessive production of ROS leads to neuronal damage in the hippocampus, producing altered Aβ peptide kinetics, leading to increased Aβ load, and contributing to AD progression, thereby causing oxidative damage through neurotoxic mechanisms and leading to impaired cognitive and memory function [112]. Zhao *et al.* found that in addition to scavenging superoxide anions, AP also increased the antioxidant capacity of the brain [38]. AP can stimulate the expression of critical genes involved in mitochondrial biogenesis and adenosine triphosphate (ATP) synthesis, thereby alleviating muscle atrophy in aged mice [113] and preventing hippocampal neuronal loss in rats [114]. Furthermore, apigenin enhances mitochondrial sirtuin-3 (Sirt3) activity and ATP production by inducing the master regulator of mitochondrial biogenesis, peroxisome proliferator-activated receptor γ coactivator factor-1 α (PGC-1α), as well as mitochondrial transcription factor A (TFAM) and fusion proteins to maintain normal mitochondrial characteristics [49].

During neurodegeneration, unsaturated fatty acids in neuronal cell membranes are constantly oxidized, generating large amounts of free radicals and producing lipid peroxides [115]. Among them, MDA is the most toxic metabolite, which interferes with normal neuronal metabolism and function. The body has systems for scavenging free radicals, including SOD, GSH-Px and catalase. Therefore, inhibition of oxidative stress and promotion of free radical scavenging should be an effective treatment for AD [116]. AP supplementation significantly reduces MDA levels [51]. The radical scavenging activity of AP against hydrogen peroxide and ROS has been demonstrated in numerous studies [45, 117, 118]. It has been reported that AP exerts antioxidant effects by inhibiting ROS, as well as by inhibiting the depletion of GSH in hippocampal neurons [45, 119]. Choi *et al.* found that AP could protect HT22 neurons from endoplasmic reticulum stress-induced apoptosis by reducing ROS accumulation and mitochondrial damage [120]. In addition to scavenging superoxide anions, AP also increases the antioxidant capacity of the brain. One study reported that in AD animal models, AP can inhibit oxidative stress by directing free radical scavenging and upregulating intracellular antioxidant defenses [40]. Several studies have shown that AP treatment reversed the decrease in SOD and GSH-Px activities and the increase in MDA levels, suggesting that AP has free radical scavenging activity and plays an antioxidant role in the damage response [55, 96, 121]. Oxidized hydroethidine (HEt) represents superoxide anion levels in the cerebral cortex. Oxidized HEt signals were significantly reduced in AP-treated APP/PS1 mice, as were changes in oxidative markers (*e.g*., GSH and SOD), suggesting that the AP may be involved in oxidative amelioration in the APP/PS1 mouse model [37].

Nuclear factor-erythroid 2-related factor 2 (Nrf2) is part of an important endogenous antioxidant pathway and plays a role in eliminating excess ROS [122]. AP was found to activate the Nrf2 signaling pathway through activation of amp-activated protein kinase [123]. Nicotinamide adenine dinucleotide (NAD)+ is an important metabolite associated with bioenergetics that counteracts neurodegenerative processes [124]. SIRT3 plays a critical role in controlling mitochondrial functions, such as oxidative phosphorylation, metabolism, electron transport, and oxidative stress [125, 126]. SIRT3 activity is dependent on NAD+ levels, and loss of NAD+ induces neuronal death and accelerates senescence [124, 127]. SIRT3 dysfunction leads to p53-mediated mitochondrial and neuronal damage in AD [128]. AP has been shown to maintain NAD+ levels and inhibit CD38 activity [129]. Anmedy *et al.* found that AP maintained NAD+ levels and preserved various metabolic functions, effectively maintaining a normal NAD+/NADH ratio, increasing mitochondrial SIRT3 activity, and enhancing ATP production. In turn, SIRT3 reduces the accumulation of damaged mitochondria and maintains normal mitochondrial function by enhancing their fusion or *via* mitochondrial autophagy [49].

### 4.5 Anti-apoptotic Effects

Apoptosis is an intrinsic biochemical, cellular process that regulates cell death and is essential for cell survival, homeostasis, and maintenance of optimal functional state [130]. Apoptosis regulates a number of molecular events in predetermined and programmed ways, including cell turnover, embryonic development, and immune system function, but when it is disordered, it may be the sole contributor to several diseases, including neurodegenerative manifestations [131]. Femi-Akinlosotu *et al.* reported that impairments in reference and working memory were significantly associated with neuronal cell death in the CA1 region of the hippocampus and that there was a relationship between memory impairment and neurodegenerative diseases affecting the CA1 subregion of the hippocampus [132]. AP could effectively prevent the degeneration of hippocampal neurons and maintain the normal number of intact neurons [49]. Similarly, Taupin *et al.* found that AP improved the performance of the MWM task by rodents and stimulated neurogenesis in the hippocampus of their brains [133]. AP supplementation was also shown to attenuate acrylonitrile-induced neuronal apoptosis based on a comparison of terminal deoxynucleotidyl transferase-mediated dUTP nick end labeling-positive cell counts [55].

The apoptotic genes Bcl2-associated X *(Bax)* and B-cell lymphoma-2 (*Bcl2)* are both involved in the pathogenesis of AD [134]. Among the Bcl2 family proteins, Bcl2 is anti-apoptotic, whereas Bax is pro-apoptotic [135]. Bcl2 specifically inhibits apoptosis, resisting various forms of cell death, prolonging cell life, and maintaining cell numbers [136]. The ratio of Bcl2/Bax determines, to some extent, whether apoptosis occurs [137]. AP was found to induce increased expression of Bcl2 and increase the Bcl2/Bax ratio while down-regulating apoptotic factors such as cytochrome C, Bax, Caspase 9 and Caspase 3, and exerting anti-apoptotic effects [51, 55].

Caspase 3 is a cysteine-aspartate protease that plays a key role in apoptosis and is widely used as a biomarker of apoptosis [138]. Caspase 3 is involved in the cleavage of APP and is associated with neuronal death in AD [139]. Studies have reported that AP can reduce caspase-3 and BAX levels in apoptotic neurons in early brain injury after subarachnoid hemorrhage and thereby inhibit neuronal cell death, thus exerting a neuroprotective effect [140]. Moreover, AP prevents caspase 3- and caspase 7-induced apoptosis by inhibiting the release of cytochrome C, the most important cysteine in synaptic disruption [141].

### 4.6 Regulation of the ERK/CREB/BDNF Pathway

The ERK/CREB/BDNF pathway is the most important neurotrophic pathway involved in memory and is commonly affected in AD [59]. CREB is a cellular transcription factor that plays an important role in memory formation and is activated when CREB is phosphorylated. Levels of pCREB decrease in the prefrontal cortex of patients with AD, indicating a functional disorder in CREB signal transduction [142]. Chiang *et al.* showed that AP attenuated the reduction of HSPB1 and Nrf2 and activated the TRKβ-mediated ERK signaling pathway, thereby upregulating CREB and its downstream anti-apoptotic BCL2 apoptotic regulator, thus exerting neurocytoprotective effects [143]. BDNF plays an important role in the development, regulation, and survival of basal forebrain cholinergic neurons [144]. Reduced BDNF synthesis may adversely affect cholinergic neurons in the hippocampus, cortex, and basal forebrain and may explain their selective vulnerability in AD [145]. Levels of both the precursor and mature form of BDNF and its mRNA are reduced early in AD and are associated with loss of cognitive function [146]. AP was shown to increase BDNF expression in a rat model, with concomitant cognitive improvement [147, 148]. The protective effect of AP against learning and memory impairment and its improvement of spatial memory may be related to the up-regulation of BDNF and its receptors TRKβ and pCREB [45, 51]. Concurrently, AP prevents a decrease in BDNF mRNA expression in a rat model [149]. Furthermore, AP restored the neurotrophic pathway ERK/CREB/BDNF in the cerebral cortex to improve AD-related learning and memory impairment. Therefore, AP appears to be an alternative means for the prevention and/or treatment of AD [37].

### 4.7 Promotion of Synapse Formation

The neuropathological manifestations of AD are synaptic loss, synaptic disturbance, and plasticity impairment [150]. Tzioras *et al.* found that soluble oligomers of Aβ and tau can propagate in different regions of the brain and contribute directly to synaptic dysfunction and loss [151]. It has been shown that Aβ and tau oligomers accumulate in the extracellular and synaptic cytoplasm, respectively, in areas demonstrating substantial synaptic loss, and removal of these proteins improves synaptic function [152].

Synaptic plasticity is critical for memory formation and storage. Synapsin I (SYNI) is a marker of synapse formation. Spatial learning increases SYNI mRNA and protein expression [153]. AP may play a role in improving synaptic connections and synaptic plasticity in the hippocampus [154]. Tu *et al.* found that AP increased SYNI expression, which is associated with neuroprotection and cognitive improvement [155]. Glutamate is an excitatory neurotransmitter that plays an important role in synaptic plasticity, learning, and memory [156]. However, excessive glutamate stimulation induces excitotoxicity and has been implicated in the pathological processes of AD [157]. Neuroprotective effects of glutamate receptor antagonists have been reported in the literature [158]. Chang *et al.* suggested that AP may exert its presynaptic inhibitory effects by reducing Cav2.2 (N-type) and Cav2.1 (P/Q-type) channel-mediated Ca^2+^ entry, thereby inhibiting glutamate release from rat hippocampal nerve endings [159].

### 4.8 Restoration of Histone Acetylation

Histone acetylation is associated with memory formation and cognitive function under physiological and pathological conditions [160]. Furthermore, previous studies have supported the important role of histone acetylation in memory consolidation, particularly in spatial memory reconsolidation in mice [161]. The dynamic balance of histone acetylation is controlled by the competitive regulation of histone deacetylases (HDACs) and histone acetylases (HATs) [162]. HDAC inhibitors reverse spatial learning and memory deficits in established mouse models of AD and activate the transcription of synaptic plasticity markers [163]. McAlpin *et al.* found that treatment with HDAC inhibitors improved long-term memory in the performance of hippocampal-dependent paradigms, such as the MWM task [164].

AP induces acetylation of H3K9 and H4K12 by inhibiting HDAC activity and decreasing the expression of HDAC1 and HDAC3 proteins in an *in vitro* study [165]. AP not only restored normal levels of histone acetylation and BDNF signaling cascades but also suppressed neuroinflammation by inhibiting pro-inflammatory cytokines and the NF-κB signaling pathway [50]. Additionally, AP inhibited HDAC expression and restored acetylated H3 and H4 levels, promoted BDNF and SYNI expression, and facilitated synaptic protein expression in the ischemic hippocampus [155].

### 4.9 Inhibition of AChE

The cholinergic hypothesis, which was first proposed more than 20 years ago, suggests that dysfunction of acetylcholine neurons in the brain is largely responsible for cognitive decline in older adults and AD patients [166]. Neurodegeneration in AD can affect many types of neurons, particularly those in the cortex and hippocampus. Among the various neurotransmitter systems affected, the effect on the cholinergic system cannot be ignored [167]. AP has been reported to increase acetylcholine levels in the brain [141]. AP improves cholinergic transmission, protects the strength and integrity of the blood-brain barrier, prevents Aβ-induced decrease in acetylcholine secretion, and promotes choline uptake in different parts of the hippocampus [48]. Liu *et al.* studied the mechanism by which AP improves memory impairment in animal models. The results showed that the anti-AD and neuroprotective effects of AP on Aβ25-35-induced mice were related to the improvement of the cholinergic system and that AP improved learning and memory in rats by inhibiting AChE activity and increasing cortical acetylcholine level [45].

### 4.10 Inhibition of Nitrosylation

Normal levels of nitric oxide (NO) promote memory and learning, but excess NO accumulates with age and is harmful to synapses [168]. In the CNS, excess NO can produce a nitrosative stress response, leading to neurodegenerative damage [169, 170]. In AD, oligomerization of Aβ, excessive neuroexcitation, and neuroinflammation can lead to NO production and subsequent S-nitrosylation [171]. Abnormal S-nitrosylation may lead to protein misfolding, synaptic damage, and apoptosis [172]. AP has been found to exhibit potent anti-inflammatory activity by inhibiting NO production and expression of inducible nitric oxide synthase and cyclooxygenase-2 [173]. Using an AD model derived from human induced pluripotent stem cells (iPSC), Balez *et al.* found that AP has potent cytoprotective properties that protect neuronal synapses and cell viability by promoting an overall downregulation of cytokine and NO release in inflammatory cells, thereby reducing neuronal hyperexcitability and apoptosis [141].

## 5 TOXICITY AND SAFETY

AP is becoming increasingly important as a health enhancer. It has long been considered safe, with low levels of toxicity, and appears to cross the blood-brain barrier [174]. Its intrinsic toxicity to normal and cancer cells is lower than that of other structurally related flavonoids [175, 176]. Hemolysis tests showed that the hemolysis percentage of AP was lower than that of the allowable limit of 5% after treatment for 30 min. Blood compatibility indicates that AP is safe for intravenous administration and that AP is non-toxic in mammalian systems [177]. Nevertheless, AP can induce muscle relaxation and sedation at high doses [178]. AP induces oxidative stress through different pathways, and high doses of AP may cause hepatotoxic oxidative stress-induced liver damage, possibly due to the regulation of several genes by AP at higher doses, at least in Swiss mice [179, 180]. It is difficult to achieve the therapeutic doses of AP used in clinical trials by dietary intake, and no harm has been reported to date. However, as consumers intentionally increase their AP intake from dietary supplements or pharmaceutical sources, safety issues related to higher levels of AP exposure may arise.

## 6 LIMITATIONS

Studies to date have shown that AP has promising pharmacological effects and research prospects in the treatment of cognitive dysfunction. AP can significantly improve cognitive function and memory impairment in animal models and also protects against different neurotoxin-induced neuronal damage through various mechanisms such as anti-inflammation and antioxidation, providing a new avenue for the development of anti-AD drugs. However, much needs to be done before AP can be used as a clinical drug for the treatment of AD.

The 13 studies included in this review all had control groups that were scientifically sound and enhanced the credibility of the results. While our analysis showed that AP produced favorable results in animal models of AD, some limitations remain due to the number and quality of studies included. First, we searched only the literature in four electronic databases (*e.g*., PubMed, Web of Science, EMBASE, and Cochrane Library), which may have led to some missed articles or publication bias. Relevant data were lacking in some cases, which may have had an impact on the results. Second, the literature included in this analysis was generally of low quality, and it was not possible to determine whether the correct randomization method was used and whether allocation concealment was performed. Third, the small sample size of the literature included in this study, all of which had positive results, could also have led to bias. The ultimate aim of animal trials is to translate the findings to the clinic; therefore, the conclusions reached in this analysis need to be validated by well-designed, randomized, controlled clinical trials. Finally, the pharmacological mechanisms of AP and AD have not been extensively studied, and the number of studies using AP as an intervention in AD is limited. Therefore, standardized intervention doses and timing of administration, as well as pharmacokinetic studies, are required. We hope that future studies will address these deficiencies.

## CONCLUSION

In this systematic review and meta-analysis, we comprehensively examined 13 studies investigating the effect of AP on animal models of AD. Our results indicated that AP is a promising candidate for AD treatment, as it appeared to improve cognitive impairment in animal models of AD and alleviate neuropathological features in the brain. We also addressed possible mechanisms underlying the neuroprotective effects of AP. Furthermore, we highlighted the limitations of current preclinical studies of AP, such as poor methodological quality, a high degree of bias, and insufficient mechanistic exploration. This information may guide the design of further experiments and clinical trials when appropriate. However, our results clearly indicate that AP is an avenue worth exploring as a treatment for AD.

## Figures and Tables

**Fig. (2):**
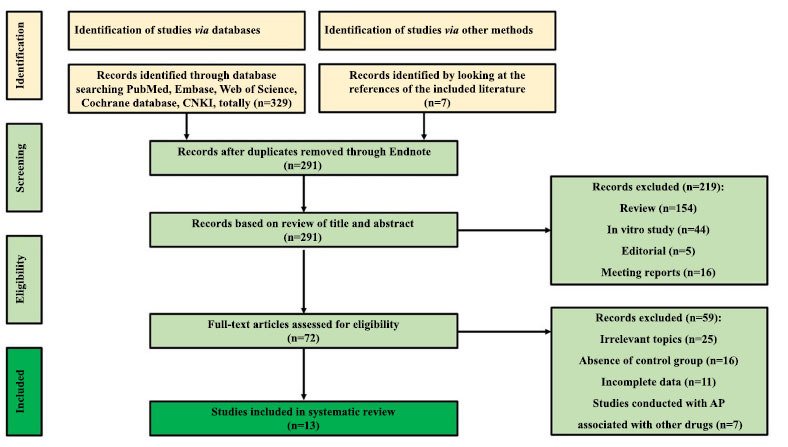
Flow diagram of the systematic review. The process of paper inclusion was divided into three steps: searching, de-duplication, and manual screening.

**Table 1:** Inclusive study on the neuroprotective effect of apigenin on Alzheimer's disease.

**Study (Year)**	**Species (Sex, Amount)**	**Weight (g)**	**Model**	**Intervention Group** **(Dose, Time, Methods)**
Ahmedy *et al*., 2022	Swiss albino mouse, male, n = 72	25-30 g	LPS	AP (40 mg/kg) for 7 days, oral
Alsadat *et al*., 2021	Wistar rat, male, n = 50	200-250 g	Aβ_25-35_	AP (50 mg/kg) for 4 weeks, oral
Chen *et al*., 2017	SD rat, male, n = 100	500-600 g	ISO	AP (25/50/100 mg/kg) for 7 days, i.p
Fan *et al*., 2023	Wistar rat, male, n = 100	220-250 g	Aβ1_-42_	AP (20/40 mg/kg) for 28 days, i.p
Jameie *et al*., 2021	Wistar rat, female, n = 42	200-250 g	OVX	AP (2 mg) for 5 weeks, i.p
Kim *et al*., 2021	ICR mice, male, n = 50	25-30 g	SCOP	AP (10/20 mg/kg) for 14 days, oral
Liu *et al*., 2011	Kunming mouse, male, n = 40	25-30 g	Aβ_25-35_	AP (10/20 mg/kg) for 8 days, oral
Mao *et al*., 2015	Wistar rat, male, n = 40	240 ± 10 g	STZ	AP (10/20/40 mg/kg) for 21 days, i.p
Nikbakht *et al*., 2019	Wistar rat, male, n = 50	200-250 g	Aβ_25-35_	AP (50 mg/kg) for 4 weeks, oral
Sang *et al*., 2017	Kunming mouse, male, n = 36	20 ± 2 g	D-galactose	AP (50 mg/kg) for 8 weeks, oral
Taha *et al*., 2017	SD rat, male, n = 60	150-200 g	MTX	AP (20 mg/kg) for 30 days, oral
Zhao *et al*., 2013	B6C3-Tg and wild-type mice, male and female, n = 36	NA	APP/PS1	AP (40 mg/kg) for 12 weeks, oral
Zhao *et al*., 2019	SD rats, male, n = 60	180-220 g	ACN	AP (117/234/351 mg/kg) for 28 days, oral

**Abbreviations**: Aβ: amyloid β-peptide; ACN: acetonitrile; APP/PS1: amyloid precursor protein/presenilin 1; ICR: imprinting control region; i.p.: intraperitoneal; ISO: isoflurane; LPS: lipo-polysaccharide; MCAO: middle cerebral artery occlusion; OVX: ovariectomize; SCOP: scopolamine; SD: Sprague-Dawley.

**Table 2:** Main outcome indexes and conclusions of apigenin in the treatment of Alzheimer's disease.

**Study (Year)**	**Outcome**	**Main Conclusion**
Ahmedy *et al*., 2022	MWM, Y-type maze alternates spontaneously, ATP, PGC-1α, PGC-1α, TFAM, eMFN2, MFN2, OPA1, PINK-1, Parkin, LC3II/I, NAD+/NADH, SIRT3, Intact neurons count in CA1 and CA3	AP has significant neuroprotective potential against lps-induced neurotoxicity by inhibiting NAD^+^ depletion and activating SIRT3 to maintain adequate mitochondrial homeostasis and function.
Alsadat *et al*., 2021	FJB positive neurons in the CA3 area, BACE1 mRNA, p-tau protein, p-tau-positive cells, GSK-3β, GSK-3α	AP has a protective effect on Aβ25-35 by reducing the hyperphosphorylation of tau and inhibiting the expression of BACE1, which in turn reduces the expression of GSK-3β.
Chen *et al*., 2017	MWM, H3K9, H4K12, HDAC1, HDAC2, CBP, Pro BDNF, BDNF, TrKB, p-CAMKII, p-CREB, p-ERK, IL-2, IL-4, IL-10, IκBα, NF-κB p65	AP restores cognitive function by restoring histone acetylation and inhibiting neuroinflammation.
Fan *et al*., 2023	MWM, Damaged neurons in the CA1 region, SOD, GSH-Px, MDA, TNF-α, IL-1β, IL-6, Nrf2, HO-1, NF-κB p65	Apigenin can inhibit oxidative stress and inflammatory response in hippocampal tissue of AD rats caused by Aβ1-42 and improve AD symptoms, and its effect may be related to activation of Nrf2/HO-1 pathway and inhibition of NF-κB nuclear translocation.
Jameie *et al*., 2021	β-Amyloid plaque count, Live and necrotic neurons in the hippocampus, MWM, Caspase-3	AP and β-estradiol significantly reduced the number of β-amyloid plaque symptoms of memory and learning disorders and decreased the expression of Caspase-3.
Kim *et al*., 2021	T-Maze Test, Novel Object Recognition Test, MWM, MDA, Bax/Bcl-2, Cleaved caspase-3, PARP, BACE, PS1, PS2, IDE, RAGE, BDNF, TrkB	AP is an active substance that can improve cognitive and memory function by regulating apoptosis, amyloid production and BDNF/TrkB signaling pathway.
Liu *et al*., 2011	MWM, rCBF, TrkB, pCREB, BDNF, ACh, AChE	Oral AP has robust neurovascular coupling protection against Aβ25-35-induced amnesia in mice, including improved learning and memory, improved cholinergic system, including inhibition of AChE activity and increased ACh levels, modified BNDF, TrkB, and p-CREB levels.
Mao *et al*., 2015	MWM, MDA, SOD, GSH, cNOS, iNOS, Caspase-3, Caspase-9	AP attenuates DACD in rats by suppressing oxidative stress, nitric oxide and apoptotic cascades synthase pathway.
Nikbakht *et al*., 2019	Y maze test, Neuronal loss was detected in the hilar area of the hippocampus, Cytochrome c positive cells and Caspase-9 positive cells.	AP may ameliorate spatial working memory impairment and neuronal degeneration by improving mitochondrial dysfunction.
Sang *et al*., 2017	OFT, Rota-rod test, MWM, AGEs, SOD, CAT, MDA, Nrf2, HO-1, NQO1	AP may play an anti-aging role by activating the NRF2 pathway.
Taha *et al*., 2017	MWM, ROCK-1, ERK1/2, CREB, BDNF, GSH, MDA, Caspase-3, IL-1β, Intact neurons count in DG, Intact neurons count in CA3 area	AP lessens MTX-induced neuroinflammation, oxidative stress, and apoptosis and boosts cognitive function by inhibiting microglial activation *via* modulating the miR-15a/ROCK-1/ERK1/2/CREB/BDNF pathway.
Zhao *et al*., 2013	MWM, Aβ, BACE1, flAPP, β-CFT, SOD, GSH-Px, BDNF, p-ERK/ERK, p-CREB/CREB	AP may ameliorate AD-related learning and memory impairment by reducing Aβ burden, inhibiting the process of amyloidosis, inhibiting oxidative stress and restoring the ERK/CREB/BDNF pathway.
Zhao *et al*., 2019	OFT, GSH, SOD, GSH-Px, HMGB-1, TLR4, IKK-α, p-IKK-α/β, IκB-α, p-IκB-α, NF-κB p65, IL-6, IL-1β, TNF-α, TUNEL-positive cells, Cyt-c, Bax, Bcl-2, Caspase-9, Caspase-3	AP can protect the brain from ACN-induced neurotoxicity by inhibiting the TLR4/ NF-κB signaling pathway.

**Abbreviations**: Aβ: amyloid β-peptide; Ach: acetylcholine; AChE: acetylcholinesterase; ACN: acetonitrile; AGEs: advanced glycation end-products; AP: apigenin; ATP: adenosine triphosphate; BACE-1: β-secreatase; Bax: Bcl2-associated x; Bcl-2: B-cell lymphoma-2; BDNF: brain-derived neurotrophic factor; CAMKII: calcium/calmodulin-dependent protein kinase II; Caspase3: cysteinyl aspartate specific proteinase; CAT: Catalase; CBP: continuous blood purification; CREB: cAMP-response element binding protein; ERK: extracellular regulated protein kinases; FJB: Fluoro-Jade B; GSH: glutathione; GSK: glycogen synthase kinase; HDAC: histone deacetylases; HMGB-1: High Mobility Group Box 1; HO-1: heme oxygenase-1; IDE: insulin-degrading enzyme; IκBα: Inhibitory Subunit of NF Kappa B Alpha; IKK-α: I Kappa B Kinase Alpha; IL-1β: interleukin-1β; iNOS: inducible NO synthase; LC3II/I: light chain; LPS: Lipo-polysaccharide; MDA: malondialdehyde; MFN2: Mitofusin-2; NAD^+^: nicotinamide adenine dinucleotide; NF-κB: nuclear factor-kappa B; NQO1,NAD(P)H quinone dehydrogenase 1; NRF2: nuclear factor erythroid 2-related factor 2; OPA1: optic atrophy protein 1; OFT: Open field test; PARP: poly(ADP-ribose) polymerase; pCREB: phosphorylated cAMP response element binding protein; PGC-1α: PPARγ coactivator factor-1 α; PINK-1: PTEN induced putative kinase 1; PS1: presenilin-1; RAGE: Receptor For Advanced Glycation End; rCBF: Regional Cerebral Blood Flow; ROCK-1: Rho-associated kinase; SIRT3: silent mating type information regulation 2 homolog-3; SOD: superoxide dismutase; TFAM: Recombinant Transcription Factor A: Mitochondrial; TLR4: Toll-like receptor 4; TNF-α,tumor necrosis factor-α; TrkB: Tyrosine kinase receptor B.

**Table 3:** (A) Meta-regression analysis on the results of the escape latency.

**_ES**	**Coefficient**	**Std. Err.**	***t***	***P*> | t |**	**[95% Conf. Interval]**	
Species	-1.276769	1.308573	-0.98	0.352	-4.192451	1.638913
Dose	0.7326276	1.120006	0.65	0.528	-1.762902	3.228157
_cons	-0.7197117	2.572467	-0.28	0.785	-6.451524	5.012101

**Table 3:** (B) Meta-regression analysis on the results of the percentage of time in the target quadrant.

**_ES**	**Coefficient**	**Std. Err.**	***t***	***P*> | t |**	**[95% Conf. Interval]**	
Dose	1.347005	1.242461	1.08	0.301	-1.387634	4.081644
Means	-1.735798	1.05919	-1.64	0.130	-4.067059	0.5954623
_cons	2.105522	2.407097	0.87	0.400	-3.192462	7.403506

## LIST OF ABBREVIATIONS

AChE: Acetylcholinesterase
AD: Alzheimer's Disease
AMPK: AMP-activated Protein Kinase
APP: Amyloid-beta Precursor Protein
ATP: Adenosine Triphosphate
Aβ: Amyloid β-protein
BACE1: Beta-site Amyloid Precursor Protein Cleaving Enzyme 1
BDNF: Brain-derived Neurotrophic Factor
CAT: Catalase
CI: Confidence Interval
CNS: Central Nervous System
COX-2: Cyclooxygenase-2
ERK: Extracellular Signal-regulated Kinase
GFAP: Glial Fibrillary Acidic Protein
GSH-Px: Glutathione Peroxidase
GSK3: Glycogen Synthase Kinase 3
HATs: Histone Acetylases
HDACs: Histone Deacetylases
HEt: Hydroethidine
IFN: Interferon
IL: Interleukin
iNOS: Inducible Nitric Oxide Synthase
iPSC: Induced Pluripotent Stem Cells
JNK: c-Jun N-terminal Kinase
LPS: Lipopolysaccharide
MAPK: Mitogen-activated Protein Kinase
MDA: Malondialdehyde
MWM: Maurice Water Maze
NAD: Nicotinamide Adenine Dinucleotide
NF-κB: Nuclear Factor Kappa-B
NFT: Neurofibrillary Tangles
NGF: Nerve Growth Factor
NO: Nitric Oxide
Nrf2: Nuclear Factor-erythroid 2-related Factor 2
NT: Neurotrophic Factors
pCREB: Phosphorylated cAMP Response-element Binding Protein
PGC-1α: PPARγ Coactivator Factor-1 α
PPARγ: Peroxisome Proliferator-activated Receptor γ
PRISMA: Preferred Reporting Items for Systematic Evaluations and Meta-analyses
PSCI: Post Stroke Cognitive Impairment
ROS: Reactive Oxygen Species
SD: Standard Deviations
SMD: Standardized Mean Difference
SOD: Superoxide Dismutase
STAT1: Signal Transduction And Transcriptional Activator 1
SynI: Synapsin I
TLR4: Toll-like Receptor 4
TNF: Tumor Necrosis Factor
TrKB: Tropomysin Related Kinase B

## STANDARDS OF REPORTING

PRISMA guidelines were followed.

## SUPPLEMENTARY MATERIAL

PRISMA checklist is available as supplementary material on the publisher’s website along with the published article.

## References

[1] Goldfarb D., Sheard S., Shaughnessy L., Atri A. (2019). Disclosure of
alzheimer’s disease and dementia.. J. Clin. Psychiatry,.

[2] Soria Lopez J.A., González H.M., Léger G.C. (2019). Alzheimer’s disease.. Handb. Clin. Neurol..

[3] Benmelouka A.Y., Ouerdane Y., Outani O., Alnasser Y.T., Alghamdi B.S., Perveen A., Ashraf G.M., Ebada M.A. (2022). Alzheimer’s disease-related psychosis: An overview of clinical manifestations, pathogenesis, and current treatment.. Curr. Alzheimer Res..

[4] Yang A., Wu J., Chen Y., Shen R., Kou X. (2022). Study on multi-target synergistic treatment of alzheimer’s disease based on metal chelators.. Curr. Drug Targets.

[5] Shah H., Albanese E., Duggan C., Rudan I., Langa K.M., Carrillo M.C., Chan K.Y., Joanette Y., Prince M., Rossor M., Saxena S., Snyder H.M., Sperling R., Varghese M., Wang H., Wortmann M., Dua T. (2016). Research priorities to reduce the global burden of dementia by 2025.. Lancet Neurol..

[6] Rostagno A.A. (2022). Pathogenesis of alzheimer’s disease.. Int. J. Mol. Sci..

[7] Calabrese V., Giordano J., Signorile A., Laura Ontario M., Castorina S., De Pasquale C., Eckert G., Calabrese E.J. (2016). Major pathogenic mechanisms in vascular dementia: Roles of cellular stress response and hormesis in neuroprotection.. J. Neurosci. Res..

[8] Long J.M., Holtzman D.M. (2019). Alzheimer disease: An update on pathobiology and treatment strategies.. Cell.

[9] Lane C.A., Hardy J., Schott J.M. (2018). Alzheimer’s disease.. Eur. J. Neurol..

[10] Milà-Alomà M., Ashton N.J., Shekari M., Salvadó G., Ortiz-Romero P., Montoliu-Gaya L., Benedet A.L., Karikari T.K., Lantero-Rodriguez J., Vanmechelen E., Day T.A., González-Escalante A., Sánchez-Benavides G., Minguillon C., Fauria K., Molinuevo J.L., Dage J.L., Zetterberg H., Gispert J.D., Suárez-Calvet M., Blennow K. (2022). Plasma p-tau231 and p-tau217 as state markers of amyloid-β pathology in preclinical Alzheimer’s disease.. Nat. Med..

[11] Mukhopadhyay S., Banerjee D. (2021). A primer on the evolution of aducanumab: The first antibody approved for treatment of alzheimer’s disease.. J. Alzheimers Dis..

[12] Knopman D.S., Jones D.T., Greicius M.D. (2021). Failure to demonstrate efficacy of aducanumab: An analysis of the EMERGE and ENGAGE trials as reported by Biogen, December 2019.. Alzheimers Dement..

[13] Cummings J., Rabinovici G.D., Atri A., Aisen P., Apostolova L.G., Hendrix S., Sabbagh M., Selkoe D., Weiner M., Salloway S. (2022). Aducanumab: Appropriate use recommendations update.. J. Prev. Alzheimers Dis..

[14] Dhadda S., Kanekiyo M., Li D., Swanson C.J., Irizarry M., Berry S., Kramer L.D., Berry D.A. (2022). Consistency of efficacy results across various clinical measures and statistical methods in the lecanemab phase 2 trial of early Alzheimer’s disease.. Alzheimers Res. Ther..

[15] Swanson C.J., Zhang Y., Dhadda S., Wang J., Kaplow J., Lai R.Y.K., Lannfelt L., Bradley H., Rabe M., Koyama A., Reyderman L., Berry D.A., Berry S., Gordon R., Kramer L.D., Cummings J.L. (2021). A randomized, double-blind, phase 2b proof-of-concept clinical trial in early Alzheimer’s disease with lecanemab, an anti-Aβ protofibril antibody.. Alzheimers Res. Ther..

[16] van Dyck C.H., Swanson C.J., Aisen P., Bateman R.J., Chen C., Gee M., Kanekiyo M., Li D., Reyderman L., Cohen S., Froelich L., Katayama S., Sabbagh M., Vellas B., Watson D., Dhadda S., Irizarry M., Kramer L.D., Iwatsubo T. (2023). Lecanemab in early alzheimer’s disease.. N. Engl. J. Med..

[17] Marucci G., Buccioni M., Ben D.D., Lambertucci C., Volpini R., Amenta F. (2021). Efficacy of acetylcholinesterase inhibitors in Alzheimer’s disease.. Neuropharmacology.

[18] Cummings J.L., Tong G., Ballard C. (2019). Treatment combinations for alzheimer’s disease: Current and future pharmacotherapy options.. J. Alzheimers Dis..

[19] Mir R.H., Shah A.J., Mohi-Ud-Din R., Pottoo F.H., Dar M.A., Jachak S.M., Masoodi M.H. (2021). Natural anti-inflammatory compounds as drug candidates in alzheimers disease.. Curr. Med. Chem..

[20] Taylor E., Kim Y., Zhang K., Chau L., Nguyen B.C., Rayalam S., Wang X. (2022). Antiaging mechanism of natural compounds: Effects on autophagy and oxidative stress.. Molecules.

[21] Ferreira J.P.S., Albuquerque H.M.T., Cardoso S.M., Silva A.M.S., Silva V.L.M. (2021). Dual-target compounds for Alzheimer’s disease: Natural and synthetic AChE and BACE-1 dual-inhibitors and their structure-activity relationship (SAR).. Eur. J. Med. Chem..

[22] Román G.C., Jackson R.E., Gadhia R., Román A.N., Reis J. (2019). Mediterranean diet: The role of long-chain ω-3 fatty acids in fish; polyphenols in fruits, vegetables, cereals, coffee, tea, cacao and wine; probiotics and vitamins in prevention of stroke, age-related cognitive decline, and Alzheimer disease.. Rev. Neurol..

[23] Kola A., Valensin D., Dudek D. (2021). Metal complexation mechanisms of polyphenols associated to alzheimer’s disease.. Curr. Med. Chem..

[24] Dabeek W.M., Marra M.V. (2019). Dietary quercetin and kaempferol: Bioavailability and potential cardiovascular-related bioactivity in humans.. Nutrients.

[25] Shimazu R., Anada M., Miyaguchi A., Nomi Y., Matsumoto H. (2021). Evaluation of blood-brain barrier permeability of polyphenols, anthocyanins, and their metabolites.. J. Agric. Food Chem..

[26] Salehi B., Venditti A., Sharifi-Rad M., Kręgiel D., Sharifi-Rad J., Durazzo A., Lucarini M., Santini A., Souto E., Novellino E., Antolak H., Azzini E., Setzer W., Martins N. (2019). The therapeutic potential of apigenin.. Int. J. Mol. Sci..

[27] Wang M., Firrman J., Liu L., Yam K. (2019). A review on flavonoid apigenin: Dietary intake, ADME, antimicrobial effects, and interactions with human gut microbiota.. BioMed Res. Int..

[28] Kooti W., Daraei N. (2017). A review of the antioxidant activity of celery (Apium graveolens L).. J. Evid. Based Complementary Altern. Med..

[29] Shankar E., Goel A., Gupta K., Gupta S. (2017). Plant flavone apigenin: An emerging anticancer agent.. Curr. Pharmacol. Rep..

[30] Adel M., Zahmatkeshan M., Akbarzadeh A., Rabiee N., Ahmadi S., Keyhanvar P., Rezayat S.M., Seifalian A.M. (2022). Chemotherapeutic effects of Apigenin in breast cancer: Preclinical evidence and molecular mechanisms; enhanced bioavailability by nanoparticles.. Biotechnol. Rep..

[31] Zhang Z., Cui C., Wei F., Lv H. (2017). Improved solubility and oral bioavailability of apigenin *via* Soluplus/Pluronic F127 binary mixed micelles system.. Drug Dev. Ind. Pharm..

[32] Sen K., Banerjee S., Mandal M. (2019). Dual drug loaded liposome bearing apigenin and 5-Fluorouracil for synergistic therapeutic efficacy in colorectal cancer.. Colloids Surf. B Biointerfaces.

[33] Xu R., Jiang C., Zhou L., Li B., Hu Y., Guo Y., Xiao X., Lu S. (2022). Fabrication of stable apigenin nanosuspension with peg 400 as antisolvent for enhancing the solubility and bioavailability.. AAPS PharmSciTech.

[34] Wang X., Li J., Zhao D., Li J. (2022). |Therapeutic and preventive effects of apigenin in cerebral ischemia: A review.. Food Funct..

[35] Chuang C.M., Monie A., Wu A., Hung C.F. (2009). Combination of apigenin treatment with therapeutic HPV DNA vaccination generates enhanced therapeutic antitumor effects.. J. Biomed. Sci..

[36] Rahimi A., Alimohammadi M., Faramarzi F., Alizadeh-Navaei R., Rafiei A. (2022). The effects of apigenin administration on the inhibition of inflammatory responses and oxidative stress in the lung injury models: A systematic review and meta-analysis of preclinical evidence.. Inflammopharmacology.

[37] Zhao L., Wang J.L., Liu R., Li X.X., Li J.F., Zhang L. (2013). Neuroprotective, anti-amyloidogenic and neurotrophic effects of apigenin in an Alzheimer’s disease mouse model.. Molecules.

[38] Zhao L., Wang J., Wang Y., Fa X. (2013). Apigenin attenuates copper-mediated β-amyloid neurotoxicity through antioxidation, mitochondrion protection and MAPK signal inactivation in an AD cell model.. Brain Res..

[39] de Font-Réaulx Rojas E., Dorazco-Barragan G. (2010). [Clinical stabilisation in neurodegenerative diseases: Clinical study in phase II. Rev. Neurol..

[40] Münch G., Venigalla M., Sonego S., Gyengesi E. (2015). Curcumin and Apigenin - novel and promising therapeutics against chronic neuroinflammation in Alzheimer′s disease.. Neural Regen. Res..

[41] Salameh J.P., Bossuyt P.M., McGrath T.A., Thombs B.D., Hyde C.J., Macaskill P., Deeks J.J., Leeflang M., Korevaar D.A., Whiting P., Takwoingi Y., Reitsma J.B., Cohen J.F., Frank R.A., Hunt H.A., Hooft L., Rutjes A.W.S., Willis B.H., Gatsonis C., Levis B., Moher D., McInnes M.D.F. (2020). Preferred reporting items for systematic review and meta-analysis of diagnostic test accuracy studies (PRISMA-DTA): Explanation, elaboration, and checklist.. BMJ.

[42] Hooijmans C.R., Rovers M.M., de Vries R.B.M., Leenaars M., Ritskes-Hoitinga M., Langendam M.W. (2014). SYRCLE’s risk of bias tool for animal studies.. BMC Med. Res. Methodol..

[43] Yamaura K., Nelson A.L., Nishimura H., Rutledge J.C., Ravuri S.K., Bahney C., Philippon M.J., Huard J. (2023). The effects of losartan or angiotensin II receptor antagonists on cartilage: A systematic review.. Osteoarthritis Cartilage.

[44] Alsadat A.M., Nikbakht F., Hossein Nia H., Golab F., Khadem Y., Barati M., Vazifekhah S. (2021). GSK-3β as a target for apigenin-induced neuroprotection against Aβ 25-35 in a rat model of Alzheimer’s disease.. Neuropeptides.

[45] Liu R., Zhang T., Yang H., Lan X., Ying J., Du G. (2011). The flavonoid apigenin protects brain neurovascular coupling against amyloid-beta(2)(5)(-)(3)(5)-induced toxicity in mice.. J. Alzheimers Dis..

[46] Nikbakht F., Khadem Y., Haghani S., Hoseininia H., Moein Sadat A., Heshemi P., Jamali N. (2019). Protective role of apigenin against Aβ 25-35 toxicity *via* inhibition of mitochondrial cytochrome c release.. Basic Clin. Neurosci..

[47] Fan H., Kang K., Li Z. (2023). Effects of apigenin on oxidative stress and inflammatory reaction in hippocampus of rats with alzheimer’s disease induced by Aβ 1 - 42. Acta.. Chinese. Med..

[48] Jameie S.B., Pirasteh A., Naseri A., Jameie M.S., Farhadi M., Babaee J.F., Elyasi L. (2021). β-amyloid formation, memory, and learning decline following long-term ovariectomy and its inhibition by systemic administration of apigenin and β-estradiol.. Basic Clin. Neurosci..

[49] Ahmedy O.A., Abdelghany T.M., El-Shamarka M.E.A., Khattab M.A., El-Tanbouly D.M. (2022). Apigenin attenuates LPS-induced neurotoxicity and cognitive impairment in mice *via* promoting mitochondrial fusion/mitophagy: Role of SIRT3/PINK1/Parkin pathway.. Psychopharmacology.

[50] Chen L., Xie W., Xie W., Zhuang W., Jiang C., Liu N. (2017). Apigenin attenuates isoflurane-induced cognitive dysfunction *via* epigenetic regulation and neuroinflammation in aged rats.. Arch. Gerontol. Geriatr..

[51] Kim Y., Kim J., He M., Lee A., Cho E. (2021). Apigenin ameliorates scopolamine-induced cognitive dysfunction and neuronal damage in mice.. Molecules.

[52] Mao X.Y., Yu J., Liu Z.Q., Zhou H.H. (2015). Apigenin attenuates diabetes-associated cognitive decline in rats *via* suppressing oxidative stress and nitric oxide synthase pathway.. Int. J. Clin. Exp. Med..

[53] Sang Y., Zhang F., Wang H., Yao J., Chen R., Zhou Z., Yang K., Xie Y., Wan T., Ding H. (2017). Apigenin exhibits protective effects in a mouse model of D -galactose-induced aging *via* activating the Nrf2 pathway.. Food Funct..

[54] Taha M., Eldemerdash O.M., Elshaffei I.M., Yousef E.M., Soliman A.S., Senousy M.A. (2023). Apigenin attenuates hippocampal microglial activation and restores cognitive function in methotrexate-treated rats: Targeting the miR-15a/ROCK-1/ERK1/2 Pathway.. Mol. Neurobiol..

[55] Zhao F., Dang Y., Zhang R., Jing G., Liang W., Xie L., Li Z. (2019). Apigenin attenuates acrylonitrile-induced neuro-inflammation in rats: Involved of inactivation of the TLR4/NF-κB signaling pathway.. Int. Immunopharmacol..

[56] Tucker L.B., Velosky A.G., McCabe J.T. (2018). Applications of the Morris water maze in translational traumatic brain injury research.. Neurosci. Biobehav. Rev..

[57] Balakrishnan R., Park J.Y., Cho D.Y., Ahn J.Y., Yoo D.S., Seol S.H., Yoon S.H., Choi D.K. (2023). AD-1 small molecule improves learning and memory function in scopolamine-induced amnesic mice model through regulation of CREB/BDNF and NF-κB/MAPK signaling pathway.. Antioxidants.

[58] Mancuso C., Capone C., Ranieri S.C., Fusco S., Calabrese V., Eboli M.L., Preziosi P., Galeotti T., Pani G. (2008). Bilirubin as an endogenous modulator of neurotrophin redox signaling.. J. Neurosci. Res..

[59] Pak M.E., Yang H.J., Li W., Kim J.K., Go Y. (2022). Yuk-Gunja-Tang attenuates neuronal death and memory impairment *via* ERK/CREB/BDNF signaling in the hippocampi of experimental Alzheimer’s disease model.. Front. Pharmacol..

[60] Shadfar S., Parakh S., Jamali M.S., Atkin J.D. (2023). Redox dysregulation as a driver for DNA damage and its relationship to neurodegenerative diseases.. Transl. Neurodegener..

[61] Vanni S., Colini Baldeschi A., Zattoni M., Legname G. (2020). Brain aging: A Ianus ‐faced player between health and neurodegeneration.. J. Neurosci. Res..

[62] Obeng E. (2021). Apoptosis (programmed cell death) and its signals - A review.. Braz. J. Biol..

[63] Shen B., Chen H.B., Zhou H.G., Wu M.H. (2021). Celastrol induces caspase-dependent apoptosis of hepatocellular carcinoma cells by suppression of mammalian target of rapamycin.. J. Tradit. Chin. Med..

[64] Arnaud L., Benech P., Greetham L., Stephan D., Jimenez A., Jullien N., García-González L., Tsvetkov P.O., Devred F., Sancho-Martinez I., Izpisua Belmonte J.C., Baranger K., Rivera S., Nivet E. (2022). APOE4 drives inflammation in human astrocytes *via* TAGLN3 repression and NF-κB activation.. Cell Rep..

[65] Nafea M., Elharoun M., Abd-Alhaseeb M.M., Helmy M.W. (2023). Leflunomide abrogates neuroinflammatory changes in a rat model of Alzheimer’s disease: The role of TNF-α/NF-κB/IL-1β axis inhibition.. Naunyn Schmiedebergs Arch. Pharmacol..

[66] Moya-Alvarado G., Tiburcio-Felix R., Ibáñez M.R., Aguirre-Soto A.A., Guerra M.V., Wu C., Mobley W.C., Perlson E., Bronfman F.C. (2023). BDNF/TrkB signaling endosomes in axons coordinate CREB/mTOR activation and protein synthesis in the cell body to induce dendritic growth in cortical neurons.. eLife.

[67] Saura C.A., Cardinaux J.R. (2017). Emerging roles of CREB-regulated transcription coactivators in brain physiology and pathology.. Trends Neurosci..

[68] Bai L.L., Zhang L.Q., Ma J., Li J., Tian M., Cao R.J., He X.X., He Z.X., Yu H.L., Zhu X.J. (2021). DIP2A is involved in SOD-mediated antioxidative reactions in murine brain.. Free Radic. Biol. Med..

[69] Hanna R., Rozenberg A., Saied L., Ben-Yosef D., Lavy T., Kleifeld O. (2023). In-depth characterization of apoptosis N-terminome reveals a link between caspase-3 cleavage and posttranslational N-terminal acetylation.. Mol. Cell. Proteomics.

[70] Mishra R., Phan T., Kumar P., Morrissey Z., Gupta M., Hollands C., Shetti A., Lopez K.L., Maienschein-Cline M., Suh H., Hen R., Lazarov O. (2022). Augmenting neurogenesis rescues memory impairments in Alzheimer’s disease by restoring the memory-storing neurons.. J. Exp. Med..

[71] Luo Z., Xu H., Liu L., Ohulchanskyy T.Y., Qu J. (2021). Optical imaging of beta-amyloid plaques in alzheimer’s disease.. Biosensors.

[72] Cho Y., Bae H.G., Okun E., Arumugam T.V., Jo D.G. (2022). Physiology and pharmacology of amyloid precursor protein.. Pharmacol. Ther..

[73] Lin Y., Im H., Diem L.T., Ham S. (2019). Characterizing the structural and thermodynamic properties of Aβ42 and Aβ40.. Biochem. Biophys. Res. Commun..

[74] Trujillo-Estrada L., Vanderklish P.W., Nguyen M.M.T., Kuang R.R., Nguyen C., Huynh E., da Cunha C., Javonillo D.I., Forner S., Martini A.C., Sarraf S.T., Simmon V.F., Baglietto-Vargas D., LaFerla F.M. (2021). SPG302 reverses synaptic and cognitive deficits without altering amyloid or tau pathology in a transgenic model of alzheimer’s disease.. Neurotherapeutics.

[75] Girotra P., Behl T., Sehgal A., Singh S., Bungau S. (2022). Investigation of the molecular role of brain-derived neurotrophic factor in alzheimer’s disease.. J. Mol. Neurosci..

[76] Falcicchia C., Tozzi F., Arancio O., Watterson D.M., Origlia N. (2020). Involvement of p38 MAPK in synaptic function and dysfunction.. Int. J. Mol. Sci..

[77] Singh R., Ganeshpurkar A., Ghosh P., Pokle A.V., Kumar D., Singh R., Singh S.K., Kumar A. (2021). Classification of beta‐site amyloid precursor protein cleaving enzyme 1 inhibitors by using machine learning methods.. Chem. Biol. Drug Des..

[78] Schnöder L., Tomic I., Schwindt L., Helm D., Rettel M., Schulz-Schaeffer W., Krause E., Rettig J., Fassbender K., Liu Y. (2021). P38α‐MAPK phosphorylates Snapin and reduces Snapin‐mediated BACE1 transportation in APP‐transgenic mice.. FASEB J..

[79] Chen Y., Huang X., Zhang Y., Rockenstein E., Bu G., Golde T.E., Masliah E., Xu H. (2012). Alzheimer’s β-Secretase (BACE1) Regulates the cAMP/PKA/CREB Pathway Independently of β-Amyloid.. J. Neurosci..

[80] Li Y., Zhang J., Wan J., Liu A., Sun J. (2020). Melatonin regulates Aβ production/clearance balance and Aβ neurotoxicity: A potential therapeutic molecule for Alzheimer’s disease.. Biomed. Pharmacother..

[81] Siddique Y.H. (2022). Rahul; Ara, G.; Afzal, M.; Varshney, H.; Gaur, K.; Subhan, I.; Mantasha, I.; Shahid, M. Beneficial effects of apigenin on the transgenic Drosophila model of Alzheimer’s disease.. Chem. Biol. Interact..

[82] Zhao L., Woody S.K., Chhibber A. (2015). Estrogen receptor β in Alzheimer's
disease: From mechanisms to therapeutics. Ageing Res
Rev.

[83] Karran E., De Strooper B. (2022). The amyloid hypothesis in Alzheimer disease: New insights from new therapeutics.. Nat. Rev. Drug Discov..

[84] Lee Y.H. (2021). Im, E.; Hyun, M.; Park, J.; Chung, K.C. Protein phosphatase PPM1B inhibits DYRK1A kinase through dephosphorylation of pS258 and reduces toxic tau aggregation.. J. Biol. Chem..

[85] Baptista F.I., Henriques A.G., Silva A.M.S., Wiltfang J. (2014). da Cruz e Silva, O.A.B. Flavonoids as therapeutic compounds targeting key proteins involved in Alzheimer’s disease.. ACS Chem. Neurosci..

[86] Liu B., Liu J., Shi J.S. (2020). SAMP8 mice as a model of age-related cognition decline with underlying mechanisms in alzheimer’s disease.. J. Alzheimers Dis..

[87] Mattsson-Carlgren N., Janelidze S., Bateman R.J., Smith R., Stomrud E., Serrano G.E., Reiman E.M., Palmqvist S., Dage J.L., Beach T.G., Hansson O. (2021). Soluble P‐tau217 reflects amyloid and tau pathology and mediates the association of amyloid with tau.. EMBO Mol. Med..

[88] Gratuze M., Chen Y., Parhizkar S., Jain N., Strickland M.R., Serrano J.R., Colonna M., Ulrich J.D., Holtzman D.M. (2021). Activated microglia mitigate Aβ-associated tau seeding and spreading.. J. Exp. Med..

[89] Lauretti E., Dincer O., Praticò D. (2020). Glycogen synthase kinase-3 signaling in Alzheimer’s disease.. Biochim. Biophys. Acta Mol. Cell Res..

[90] Giovinazzo D., Bursac B., Sbodio J.I., Nalluru S., Vignane T., Snowman A.M., Albacarys L.M., Sedlak T.W., Torregrossa R., Whiteman M., Filipovic M.R., Snyder S.H., Paul B.D. (2021). Hydrogen sulfide is neuroprotective in Alzheimer’s disease by sulfhydrating GSK3β and inhibiting Tau hyperphosphorylation.. Proc. Natl. Acad. Sci..

[91] Ozben T., Ozben S. (2019). Neuro-inflammation and anti-inflammatory treatment options for Alzheimer’s disease.. Clin. Biochem..

[92] Forloni G., Balducci C. (2018). Alzheimer’s disease, oligomers, and inflammation.. J. Alzheimers Dis..

[93] Newcombe E.A., Camats-Perna J., Silva M.L., Valmas N., Huat T.J., Medeiros R. (2018). Inflammation: the link between comorbidities, genetics, and Alzheimer’s disease.. J. Neuroinflammation.

[94] Ng A., Tam W.W., Zhang M.W., Ho C.S., Husain S.F., McIntyre R.S., Ho R.C. (2018). IL-1β, IL-6, TNF- α and CRP in Elderly Patients with Depression or Alzheimer’s disease: Systematic Review and Meta-Analysis.. Sci. Rep..

[95] Al-Khayri J.M., Sahana G.R., Nagella P., Joseph B.V., Alessa F.M., Al-Mssallem M.Q. (2022). Flavonoids as potential anti-inflammatory molecules: A review.. Molecules.

[96] Zhang F., Li F., Chen G. (2014). Neuroprotective effect of apigenin in rats after contusive spinal cord injury.. Neurol. Sci..

[97] Zhang T., Su J., Guo B., Wang K., Li X., Liang G. (2015). Apigenin protects blood-brain barrier and ameliorates early brain injury by inhibiting TLR4-mediated inflammatory pathway in subarachnoid hemorrhage rats.. Int. Immunopharmacol..

[98] Hostetler G., Riedl K., Cardenas H., Diosa-Toro M., Arango D., Schwartz S., Doseff A.I. (2012). Flavone deglycosylation increases their anti‐inflammatory activity and absorption.. Mol. Nutr. Food Res..

[99] Rezai-Zadeh K., Ehrhart J., Bai Y., Sanberg P.R., Bickford P., Tan J., Shytle R.D. (2008). Apigenin and luteolin modulate microglial activation *via* inhibition of STAT1-induced CD40 expression.. J. Neuroinflammation.

[100] Bugiani M., Plug B.C., Man J.H.K., Breur M., van der Knaap M.S. (2022). Heterogeneity of white matter astrocytes in the human brain.. Acta Neuropathol..

[101] Ioannou M.S., Jackson J., Sheu S.H., Chang C.L., Weigel A.V., Liu H., Pasolli H.A., Xu C.S., Pang S., Matthies D., Hess H.F., Lippincott-Schwartz J., Liu Z. (2019). Neuron-astrocyte metabolic coupling protects against activity-induced fatty acid toxicity.. Cell.

[102] Amalia L. (2021). Glial Fibrillary Acidic Protein (GFAP): Neuroinflammation biomarker in acute ischemic stroke.. J. Inflamm. Res..

[103] Liang H., Sonego S., Gyengesi E., Rangel A., Niedermayer G., Karl T., Münch G. (2017). Anti-inflammatory and neuroprotective effect of apigenin: Studies in the gfap-il6 mouse model of chronic neuroinflammation.. Free Radic. Biol. Med..

[104] Che D.N., Cho B.O., Kim J., Shin J.Y., Kang H.J., Jang S.I. (2020). Effect of luteolin and apigenin on the production of Il-31 and Il-33 in lipopolysaccharides-activated microglia cells and their mechanism of action.. Nutrients.

[105] Ionescu-Tucker A., Cotman C.W. (2021). Emerging roles of oxidative stress in brain aging and Alzheimer’s disease.. Neurobiol. Aging.

[106] Calabrese V., Cornelius C., Dinkova-Kostova A.T., Calabrese E.J., Mattson M.P. (2010). Cellular stress responses, the hormesis paradigm, and vitagenes: Novel targets for therapeutic intervention in neurodegenerative disorders.. Antioxid. Redox Signal..

[107] Javed H., Mohamed Fizur N.M., Jha N.K., Ashraf G.M., Ojha S. (2022). Neuroprotective potential and underlying pharmacological mechanism of carvacrol for alzheimer’s and parkinson’s diseases.. Curr. Neuropharmacol..

[108] Bertholet A.M., Delerue T., Millet A.M., Moulis M.F., David C., Daloyau M., Arnauné-Pelloquin L., Davezac N., Mils V., Miquel M.C., Rojo M., Belenguer P. (2016). Mitochondrial fusion/fission dynamics in neurodegeneration and neuronal plasticity.. Neurobiol. Dis..

[109] Giorgi C., Marchi S., Pinton P. (2018). The machineries, regulation and cellular functions of mitochondrial calcium.. Nat. Rev. Mol. Cell Biol..

[110] Zhang B., Pan C., Feng C., Yan C., Yu Y., Chen Z., Guo C., Wang X. (2022). Role of mitochondrial reactive oxygen species in homeostasis regulation.. Redox Rep..

[111] Yu H., Lin X., Wang D., Zhang Z., Guo Y., Ren X., Xu B., Yuan J., Liu J., Spencer P.S., Wang J.Z., Yang X. (2018). Mitochondrial molecular abnormalities revealed by proteomic analysis of hippocampal organelles of mice triple transgenic for alzheimer disease.. Front. Mol. Neurosci..

[112] Gonzalez P., Sabater L., Mathieu E., Faller P., Hureau C. (2022). Why the ala-his-his peptide is an appropriate scaffold to remove and redox silence copper ions from the alzheimer’s-related Aβ peptide.. Biomolecules.

[113] Wang D., Yang Y., Zou X., Zhang J., Zheng Z., Wang Z. (2020). Antioxidant apigenin relieves age-related muscle atrophy by inhibiting oxidative stress and hyperactive mitophagy and apoptosis in skeletal muscle of mice.. J. Gerontol. A Biol. Sci. Med. Sci..

[114] Chirumbolo S. (2019). Is mitochondria biogenesis and neuronal loss prevention in rat hippocampus promoted by apigenin?. Basic Clin. Neurosci..

[115] Sukhorukov V.S., Mudzhiri N.M., Voronkova A.S., Baranich T.I., Glinkina V.V., Illarioshkin S.N. (2021). Mitochondrial disorders in alzheimer’s disease.. Biochemistry.

[116] Oh Y., Ahn C.B., Nam K.H., Kim Y.K., Yoon N., Je J.Y. (2019). Amino acid composition, antioxidant, and cytoprotective effect of blue mussel (mytilus edulis) hydrolysate through the inhibition of caspase-3 activation in oxidative stress-mediated endothelial cell injury.. Mar. Drugs.

[117] Lin C.M., Chen C.T., Lee H.H., Lin J.K. (2002). Prevention of cellular ROS damage by isovitexin and related flavonoids.. Planta Med..

[118] Özyürek M., Bektaşoğlu B., Güçlü K., Apak R. (2009). Measurement of xanthine oxidase inhibition activity of phenolics and flavonoids with a modified cupric reducing antioxidant capacity (CUPRAC) method.. Anal. Chim. Acta.

[119] Han J.Y., Ahn S.Y., Kim C.S., Yoo S.K., Kim S.K., Kim H.C., Hong J.T., Oh K.W. (2012). Protection of apigenin against kainate-induced excitotoxicity by anti-oxidative effects.. Biol. Pharm. Bull..

[120] Choi A.Y., Choi J.H., Lee J.Y., Yoon K.S., Choe W., Ha J., Yeo E.J., Kang I. (2010). Apigenin protects HT22 murine hippocampal neuronal cells against endoplasmic reticulum stress-induced apoptosis.. Neurochem. Int..

[121] Dou X., Zhou Z., Ren R., Xu M. (2020). Apigenin, flavonoid component isolated from Gentiana veitchiorum flower suppresses the oxidative stress through LDLR-LCAT signaling pathway.. Biomed. Pharmacother..

[122] Yang X., Fang Y., Hou J., Wang X., Li J., Li S., Zheng X., Liu Y., Zhang Z. (2022). The heart as a target for deltamethrin toxicity: Inhibition of Nrf2/HO-1 pathway induces oxidative stress and results in inflammation and apoptosis.. Chemosphere.

[123] Perez-Leal O., Barrero C.A., Merali S. (2017). Pharmacological stimulation of nuclear factor (erythroid-derived 2)-like 2 translation activates antioxidant responses.. J. Biol. Chem..

[124] Lautrup S., Sinclair D.A., Mattson M.P., Fang E.F. (2019). NAD+ in brain aging and neurodegenerative disorders.. Cell Metab..

[125] Meng H., Yan W.Y., Lei Y.H., Wan Z., Hou Y.Y., Sun L.K., Zhou J.P. (2019). SIRT3 regulation of mitochondrial quality control in neurodegenerative diseases.. Front. Aging Neurosci..

[126] Sidorova-Darmos E., Sommer R., Eubanks J.H. (2018). The role of SIRT3 in the brain under physiological and pathological conditions.. Front. Cell. Neurosci..

[127] Satoh A., Imai S., Guarente L. (2017). The brain, sirtuins, and ageing.. Nat. Rev. Neurosci..

[128] Lee J., Kim Y., Liu T., Hwang Y.J., Hyeon S.J. (2018). Im, H.; Lee, K.; Alvarez, V.E.; McKee, A.C.; Um, S.J.; Hur, M.; Mook-Jung, I.; Kowall, N.W.; Ryu, H. SIRT3 deregulation is linked to mitochondrial dysfunction in Alzheimer’s disease.. Aging Cell.

[129] Escande C., Nin V., Price N.L., Capellini V., Gomes A.P., Barbosa M.T., O’Neil L., White T.A., Sinclair D.A., Chini E.N. (2013). Flavonoid apigenin is an inhibitor of the NAD+ ase CD38: Implications for cellular NAD+ metabolism, protein acetylation, and treatment of metabolic syndrome.. Diabetes.

[130] Li R.L., Wang L.Y., Duan H.X., Zhang Q., Guo X., Wu C., Peng W. (2022). Regulation of mitochondrial dysfunction induced cell apoptosis is a potential therapeutic strategy for herbal medicine to treat neurodegenerative diseases.. Front. Pharmacol..

[131] Onyango I., Bennett J., Stokin G. (2021). Regulation of neuronal bioenergetics as a therapeutic strategy in neurodegenerative diseases.. Neural Regen. Res..

[132] Femi-Akinlosotu O.M., Shokunbi M.T., Olopade F.E., Igbong P. (2021). Deficits of learning and spatial memory are associated with increased pyknosis of pyramidal neurons of the hippocampus of adult rats with chronic hydrocephalus.. West Afr. J. Med..

[133] Taupin P. (2009). Apigenin and related compounds stimulate adult neurogenesis.. Expert Opin. Ther. Pat..

[134] Das M., Devi K.P. (2021). Dihydroactinidiolide regulates Nrf2/HO-1 expression and inhibits caspase-3/Bax pathway to protect SH-SY5Y human neuroblastoma cells from oxidative stress induced neuronal apoptosis.. Neurotoxicology.

[135] Hao Q., Chen J., Lu H., Zhou X. (2022). The ARTS of p53-dependent mitochondrial apoptosis.. J. Mol. Cell Biol..

[136] Zhang Y., Yang X., Ge X., Zhang F. (2019). Puerarin attenuates neurological deficits *via* Bcl-2/Bax/cleaved caspase-3 and Sirt3/SOD2 apoptotic pathways in subarachnoid hemorrhage mice.. Biomed. Pharmacother..

[137] Moldoveanu T., Czabotar P.E. (2020). BAX, BAK, and BOK: A Coming of Age for the BCL-2 family effector proteins.. Cold Spring Harb. Perspect. Biol..

[138] Asadi M., Taghizadeh S., Kaviani E., Vakili O., Taheri-Anganeh M., Tahamtan M., Savardashtaki A. (2022). Caspase‐3: Structure, function, and biotechnological aspects.. Biotechnol. Appl. Biochem..

[139] Zheng C., Liu S., Zhang X., Hu Y., Shang X., Zhu Z., Huang Y., Wu G., Xiao Y., Du Z., Liang Y., Chen D., Zang S., Hu Y., He M., Zhang X., Yu H. (2022). Shared genetic architecture between the two neurodegenerative diseases: Alzheimer’s disease and glaucoma.. Front. Aging Neurosci..

[140] Kim A., Nam Y.J., Lee M.S., Shin Y.K., Sohn D.S., Lee C.S. (2016). Apigenin reduces proteasome inhibition-induced neuronal apoptosis by suppressing the cell death process.. Neurochem. Res..

[141] Balez R., Steiner N., Engel M., Muñoz S.S., Lum J.S., Wu Y., Wang D., Vallotton P., Sachdev P., O’Connor M., Sidhu K., Münch G., Ooi L. (2016). Neuroprotective effects of apigenin against inflammation, neuronal excitability and apoptosis in an induced pluripotent stem cell model of Alzheimer’s disease.. Sci. Rep..

[142] Shengkai D., Qianqian L., Yazhen S. (2022). The effects and regulatory mechanism of flavonoids from stems and leaves of scutellaria baicalensis georgi in promoting neurogenesis and improving memory impairment mediated by the BDNF-ERK-CREB signaling pathway in rats.. CNS Neurol. Disord. Drug Targets.

[143] Chiang N.N., Lin T.H., Teng Y.S., Sun Y.C., Chang K.H., Lin C.Y., Hsieh-Li H.M., Su M.T., Chen C.M., Lee-Chen G.J. (2021). Flavones 7,8-DHF, quercetin, and apigenin against tau toxicity *via* activation of TRKB signaling in ΔK280 TauRD-DsRed SH-SY5Y Cells.. Front. Aging Neurosci..

[144] Orciani C., Hall H., Pentz R., Foret M.K., Do Carmo S., Cuello A.C. (2022). Long‐term nucleus basalis cholinergic depletion induces attentional deficits and impacts cortical neurons and BDNF levels without affecting the NGF synthesis.. J. Neurochem..

[145] Nguyen C.D., Yoo J., Hwang S.Y., Cho S.Y., Kim M., Jang H., No K.O., Shin J.C., Kim J.H., Lee G. (2022). Bee venom activates the Nrf2/HO-1 and TrkB/CREB/BDNF pathways in neuronal cell responses against oxidative stress induced by Aβ1-42.. Int. J. Mol. Sci..

[146] Lima Giacobbo B., Doorduin J., Klein H.C., Dierckx R.A.J.O., Bromberg E., de Vries E.F.J. (2019). Brain-derived neurotrophic factor in brain disorders: Focus on neuroinflammation.. Mol. Neurobiol..

[147] Patil S.P., Jain P.D., Sancheti J.S., Ghumatkar P.J., Tambe R., Sathaye S. (2014). RETRACTED: Neuroprotective and neurotrophic effects of Apigenin and Luteolin in MPTP induced parkinsonism in mice.. Neuropharmacology.

[148] Weng L., Guo X., Li Y., Yang X., Han Y. (2016). Apigenin reverses depression-like behavior induced by chronic corticosterone treatment in mice.. Eur. J. Pharmacol..

[149] Anusha C., Sumathi T., Joseph L.D. (2017). Protective role of apigenin on rotenone induced rat model of Parkinson’s disease: Suppression of neuroinflammation and oxidative stress mediated apoptosis.. Chem. Biol. Interact..

[150] Chen Z., Wu M., Lai Q., Zhou W., Wen X., Yin X. (2022). Epigenetic regulation of synaptic disorder in Alzheimer’s disease.. Front. Neurosci..

[151] Tzioras M., McGeachan R.I., Durrant C.S., Spires-Jones T.L. (2023). Synaptic degeneration in Alzheimer disease.. Nat. Rev. Neurol..

[152] Skaper S.D., Facci L., Zusso M., Giusti P. (2017). Synaptic plasticity, dementia and alzheimer disease.. CNS Neurol. Disord. Drug Targets.

[153] Rocchi A., Sacchetti S., De Fusco A., Giovedi S., Parisi B., Cesca F., Höltje M., Ruprecht K., Ahnert-Hilger G., Benfenati F. (2019). Autoantibodies to synapsin I sequestrate synapsin I and alter synaptic function.. Cell Death Dis..

[154] Calabrese V., Mancuso C., Calvani M., Rizzarelli E., Butterfield D.A., Giuffrida Stella A.M. (2007). Nitric oxide in the central nervous system: Neuroprotection versus neurotoxicity.. Nat. Rev. Neurosci..

[155] Tu F., Pang Q., Huang T., Zhao Y., Liu M., Chen X. (2017). Apigenin ameliorates post-stroke cognitive deficits in rats through histone acetylation-mediated neurochemical alterations.. Med. Sci. Monit..

[156] Mahato P.K., Ramsakha N., Ojha P., Gulia R., Sharma R., Bhattacharyya S., Group I. (2018). Group I metabotropic glutamate receptors (mGluRs): Ins and outs.. Adv. Exp. Med. Biol..

[157] Wang R., Reddy P.H. (2017). Role of glutamate and NMDA receptors in alzheimer’s disease.. J. Alzheimers Dis..

[158] Reglodi D., Tamas A., Jungling A., Vaczy A., Rivnyak A., Fulop B.D., Szabo E., Lubics A., Atlasz T. (2018). Protective effects of pituitary adenylate cyclase activating polypeptide against neurotoxic agents.. Neurotoxicology.

[159] Chang C.Y., Lin T.Y., Lu C.W., Wang C.C., Wang Y.C., Chou S.S.P., Wang S.J. (2015). Apigenin, a natural flavonoid, inhibits glutamate release in the rat hippocampus.. Eur. J. Pharmacol..

[160] Burns A.M., Gräff J. (2021). Cognitive epigenetic priming: leveraging histone acetylation for memory amelioration.. Curr. Opin. Neurobiol..

[161] Villain H., Florian C., Roullet P. (2016). HDAC inhibition promotes both initial consolidation and reconsolidation of spatial memory in mice.. Sci. Rep..

[162] Hyndman K.A., Knepper M.A. (2017). Dynamic regulation of lysine acetylation: The balance between acetyltransferase and deacetylase activities.. Am. J. Physiol. Renal Physiol..

[163] Dagnas M., Micheau J., Decorte L., Beracochea D., Mons N. (2015). Post‐training, intrahippocampal HDAC inhibition differentially impacts neural circuits underlying spatial memory in adult and aged mice.. Hippocampus.

[164] McAlpin B.R., Mahalingam R., Singh A.K., Dharmaraj S., Chrisikos T.T., Boukelmoune N., Kavelaars A., Heijnen C.J. (2022). HDAC6 inhibition reverses long-term doxorubicin-induced cognitive dysfunction by restoring microglia homeostasis and synaptic integrity.. Theranostics.

[165] Pandey M., Kaur P., Shukla S., Abbas A., Fu P., Gupta S. (2012). Plant flavone apigenin inhibits HDAC and remodels chromatin to induce growth arrest and apoptosis in human prostate cancer cells: In Vitro and In Vivo study.. Mol. Carcinog..

[166] de Leo G., Gulino R., Coradazzi M., Leanza G. (2022). Acetylcholine and noradrenaline differentially regulate hippocampus-dependent spatial learning and memory.. Brain Commun..

[167] Andrade S., Ramalho M.J., Pereira M.C., Loureiro J.A. (2018). Resveratrol brain delivery for neurological disorders prevention and treatment.. Front. Pharmacol..

[168] Ben-Azu B., Aderibigbe A.O., Ajayi A.M., Umukoro S., Iwalewa E.O. (2019). Involvement of L ‐arginine‐nitric oxide pathway in the antidepressant and memory promoting effects of morin in mice.. Drug Dev. Res..

[169] Lee S.Y., Lee S.J., Han C., Patkar A.A., Masand P.S., Pae C.U. (2013). Oxidative/nitrosative stress and antidepressants: Targets for novel antidepressants.. Prog. Neuropsychopharmacol. Biol. Psychiatry.

[170] Concetta Scuto M., Mancuso C., Tomasello B., Laura Ontario M., Cavallaro A., Frasca F., Maiolino L., Trovato Salinaro A., Calabrese E.J., Calabrese V. (2019). Curcumin, hormesis and the nervous system.. Nutrients.

[171] Seneviratne U., Nott A., Bhat V.B., Ravindra K.C., Wishnok J.S., Tsai L.H., Tannenbaum S.R. (2016). S -nitrosation of proteins relevant to Alzheimer’s disease during early stages of neurodegeneration.. Proc. Natl. Acad. Sci..

[172] Singh S. (2020). Updates on versatile role of putative gasotransmitter nitric oxide: Culprit in neurodegenerative disease pathology.. ACS Chem. Neurosci..

[173] Choi J.S., Nurul Islam M., Yousof Ali M., Kim E.J., Kim Y.M., Jung H.A. (2014). Effects of C-glycosylation on anti-diabetic, anti-Alzheimer’s disease and anti-inflammatory potential of apigenin.. Food Chem. Toxicol..

[174] Yang Y., Bai L., Li X., Xiong J., Xu P., Guo C., Xue M. (2014). Transport of active flavonoids, based on cytotoxicity and lipophilicity: An evaluation using the blood-brain barrier cell and Caco-2 cell models.. Toxicol. In Vitro.

[175] Ali F. (2017). Rahul; Naz, F.; Jyoti, S.; Siddique, Y.H. Health functionality of apigenin: A review.. Int. J. Food Prop..

[176] Lotha R., Sivasubramanian A. (2018). Flavonoids nutraceuticals in prevention and treatment of cancer: A review.. Asian J. Pharm. Clin. Res..

[177] Banerjee K., Banerjee S., Das S., Mandal M. (2015). Probing the potential of apigenin liposomes in enhancing bacterial membrane perturbation and integrity loss.. J. Colloid Interface Sci..

[178] Ross J.A., Kasum C.M. (2002). Dietary flavonoids: Bioavailability, metabolic effects, and safety.. Annu. Rev. Nutr..

[179] Singh P., Mishra S.K., Noel S., Sharma S., Rath S.K. (2012). Acute exposure of apigenin induces hepatotoxicity in Swiss mice.. PLoS One.

[180] Tang D., Chen K., Huang L., Li J. (2017). Pharmacokinetic properties and drug interactions of apigenin, a natural flavone.. Expert Opin. Drug Metab. Toxicol..

